# Supplementary material for: A tube-source X-ray microtomography approach for quantitative 3D microscopy of optically challenging cell-cultured samples
Source: Commun Biol. 2020 Oct 2;3:548. doi: 10.1038/s42003-020-01273-w (PMC7532209; doi:10.1038/s42003-020-01273-w)
Supplement: Supplementary file 1 — Supplementary Information [file 42003_2020_1273_MOESM1_ESM.pdf]

Supplementary Information

A tube-source X-ray microtomography approach for quantitative 3D microscopy of optically challenging cell-cultured samples

Supplementary Note 1: Introduction to advanced optical 3D imaging techniques using conventional optics

Despite their many advanced features, 3D imaging techniques using conventional optics are still limited to relatively small imaging depths. One fundamental limitation of this is set by a so-called transport mean free path (TMFP), which is the average distance in which the initial propagation direction of a photon becomes randomized due to multiple scattering events. In optimal histological samples, the TMFP of near-infrared (NIR), a less scattering portion of radiation than visible light, can reach to around 1 mm. Although most of the out-of-focus photons can be rejected by a confocal pinhole for example, beyond the TMFP the microscopic accuracy is usually lost together with the decaying signal intensity and smearing image.[1] Thus, visible spectrum 1-photon fluorescent light sheet microscopy has been demonstrated to image turbid biological samples only in the depth range of hundreds of microns[2,3]. The same axial reach applies on labeling enhanced 2-photon fluorescence used together with collagen specific NIR-excited second harmonic generation. This imaging combination has been used to study cell-seeded biomaterial scaffolds.[4] Labeling-enhanced NIR-excited 2-photon imaging alone has been demonstrated to reach 1 mm inside *ex vivo* mouse brains[5]. The imaging depth can be extended by a few hundred micrometers farther if an even less diffractive 1,675 nm excitation wavelength is combined together with a 3-photon excitation mechanism. This imaging approach has been demonstrated in the study of labeled vasculature and neurons inside mouse brains *in vivo*.[6] *In vivo* imaging of labeled mouse brain vasculature by 1,280 nm excited 2-photon fluorescence has been demonstrated at a depth of 1.6 mm, but with much smaller signal-to-background ratios and weaker lateral and axial resolutions[7]. By choosing an optimal excitation wavelength between the two previously mentioned to find a balance between the excess diffraction by tissues and strong absorption by water, and by using effective fluorescent labeling, lateral resolution of about 4 µm has been preserved down to 3 mm in mouse tissues *in vivo*. This was surprisingly demonstrated by using 1-photon fluorescence to image capillary blood vessels in brain tissue through an intact skull and scalp.[8] However, this approach is still waiting to be applied in optical sectioning to produce 3D data[9]. By using techniques referred to as optical coherence tomography, photons experiencing only one major backscattering event can also be acquired to survey the depth of reflective interfaces inside a sample. From the technical aspect, at least 10 µm lateral resolution could be preserved down to a depth of 6 mm with the technique[10]. However, to image real practical samples with both high lateral and axial resolution, 3D imaging depths of only 1.2 mm to 1.3 mm have been demonstrated[11,12]. Another potential low-energy imaging techniques are super-resolution techniques, such as structured-illumination microscopy. Super-resolution techniques are based on partial excitations of the imaged sample and the analysis of the fluorescent moiré patterns that can be used to accurately pinpoint the fluorescence labels. The super-resolution techniques can produce 3D data with accuracy beyond diffraction limit.[13] In combination with 2-photon fluorescence, super-resolution imaging has been demonstrated to exceed 3D-imaging depths of 100 µm in live tissues[14]. Thus, the reach of 3D microscopy techniques that rely on conventional optics seem to be limited to a depth below 2 mm, especially if strongly turbid samples are studied in microscopic accuracy.

Supplementary Note 2: Introduction to contrast enhancements for µCT

In µCT imaging, the most straightforward way to acquire a tomographic image is to let the X-ray photons absorb into a sample, after which the density variations and corresponding structures can be studied by assessing the shadow. This type of attenuation-based imaging is particularly straightforward to implement with inherently dense samples such as bone tissues[15]. Examples of spectacular native attenuation imaging that could be mentioned are made with sophisticated synchrotron complexes used to unveil the 3D ultrastructure of mouse cells[16] and quantify the subnuclear responses of pathogenic fungus on fungicides[17]. However, the features being studied are not always visible as such and need to be adduced in some way. Overall biological X-ray contrast can be enhanced by using general contrast agents such as iodine[18,19,20], phosphotungstic acid[19,20], or osmium tetroxide[20]. Specific antigens can be labeled by using functionalized antibodies[21,22,23]. A couple of semi-specific approaches have also been introduced to highlight isolated details without the need for antibodies. One particularly interesting approach is the enhancement of nuclei in solid tissues using hematein lead(II) complex[24]. Other approaches have been used to distinguish cells from biomaterials using general contrast agents[25,26] or surface-functionalized metal particles[27], and to trace neural pathways in brain tissues using metal impregnation[28]. Low density details may also be adduced by using so-called inline phase-contrasting, a technique that relies on the constructive and destructive phase differences of X-ray photons coming through the sample[29,30]. Thus, µCT and its well evolved contrasting toolbox form a powerful way to 3D image a wide variety of biomedical samples.

Supplementary Note 3: Introduction to µCT X-ray sources

In general, µCT technology can be divided into two performance categories based on the X-ray source used: X-ray tube-based devices[21,18,20,25,26], and synchrotrons, which are usually large scale facilities[16,17,22,27,28,30,31,32]. The former group comprises common and relatively inexpensive devices, but the far more complex and sophisticated synchrotrons produce highly brilliant and coherent X-ray beams enabling submicron resolution, short exposure times, good elemental contrast, and phase sensitivity[31]. Thus, in general, the most advanced µCT experiments such as the study of the 3D ultrastructure of mouse cells[16], the quantification of the subnuclear responses of pathogenic fungus on fungicides[17], and the 3D mapping of neural pathways in insect brains[28] are executed with the aid of synchrotron radiation. Microscopic 4D imaging of developing frog embryos has also been demonstrated with synchrotron radiation[30]. To partly fill the performance gap between X-ray tube-based devices and synchrotron technology, advances have been made to increase the imaging accuracy and source brightness in small-scale devices by decreasing the size of the radiating focal spot in nano-CT tube-sources[33] and by using a liquid-metal jet electrode tolerant to higher heat investment[34], respectively. Somewhat exotic apparatus have also been built to combine high brightness and accuracy in tabletop technologies waiting to become generalized for a wider audience. These include plasma-wakefield accelerators[35] and regenerative debris-free droplet-target laser-plasma X-ray sources[36] both capable producing high-brightness, high-quality X-rays from relative small technological ensembles. As a conclusion, low-end µCTs that are relatively small, inexpensive, and readily available, are usually limited in accuracy, image quality, and subsequent analyses when compared to more advanced X-ray systems. However, novel and useful ways to efficiently apply lower-performance technologies should be considered and experimented as shown in this paper. This way the true capabilities of pre-established tools available for the wider scientific community can be recognized and utilized. Furthermore, the same novel principles could also extend the ways how the higher-performance technologies can be used as well.

Supplementary Note 4: Main image compositions

Figure 1: Fluorescence and light microscopy images were exported as TIF images from the associated microscope software of DP manager 2.2. 1.195 and EVOS FL Auto 1202.b302b6cb2506, respectively. CLSM images were also exported as TIF files from ZEN 2011 SP3 (Black Edition) 8.1.0.484 software. The µCT image stacks were examined in Avizo 9.0.1 where Transform Sequence and Resample Transformed Image operations were used to align the imaged surfaces perpendicular with the voxel arrays. The images were then cropped to 100 µm thick slices and positioned symmetrically around the surfaces on which the cells lay. Finally, the Image Ortho Projections module was used to invert the images to resemble light microscopy, and the samples were viewed as Maximum Intensity Projections with initial linear histogram adjustments. Images in pseudo-color were turned into grayscale images and all the acquired images were appropriately scaled and paginated in Inkscape 0.48. The fluorescence and CLSM images were grayscale inverted with the Invert lightness tool. After combining the images together, if possible without causing excessive clipping of the extreme intensities, the images were normalized manually using the Lightness adjustment of the Lightness-Contrast tool.

For **Figure 2**, after reconstructions, the OPT and µCT data were visualized in Avizo 9.0.1 by Volume Rendering with volrenRed.col color map and high quality setting to show higher X-ray attenuation intensities as brighter and more opaque colors. The reconstructed OPT data was first intensity inverted to resemble the µCT view, and the lower ringing artifact was cropped away from the wet µCT FOV before final volume visualizations (an explanation of the µCT ringing artifact is given in **Supplementary Fig. 13**). To visualize the nucleus on the most protruding PLA fiber and demonstrate the constitutive intensities of these few moderately reconstructed structures, the lower and upper histogram values for the OPT data were set to 56,500 and 63,000, respectively, and 0.14 alpha value was used. For the wet µCT data, lower and upper histogram values were set to 15,200 and 18,600, respectively, and 0.96 alpha value was used. For the dry µCT data, lower and upper histogram values were set to 4,300 and 5,200, respectively, and 0.35 alpha value was used. The images were exported as PNG rgb alpha files with transparent backgrounds. All the images were appropriately scaled and paginated with black backgrounds in Inkscape 0.48. Using the same software, the OPT image was also hue inverted to give it the distinctive blue color. The PLA scaffold in **Figure 2c** was photographed next to a ruler, which was later cropped away with the other background to paginate only the scaffold itself in the correct scale with the **Figure 2b** illustrations. ZEN 2011 SP3 (Black Edition) software was used to acquire and visualize the CLSM data. For **Figure 2** volume visualizations, the Maximum projection mode was used to view the image stacks from the two different aspects, with the brighter and greener color labeling having the higher reflection intensity of the 594 nm channel used.

In **Figure 3a**, the light microscopy images were exported as TIF files from the EVOS FL Auto 1202.b302b6cb2506. All the µCT image stacks were examined in Avizo 9.0.1 where Ortho Slice and Voxelized Rendering modules were used to visualize the data in 2D and 3D, respectively. The histograms of the X-ray attenuation Ortho Slices, visualized by the grayscale.am color map, were always adjusted to Data min-max. All the 3D binary and label images were visualized by the Constant color or labelsBinary.am, and a modified labels.am color maps, respectively. In **Figure 3f**, the Spline Probe tool was used to acquire the intensity profiles along the original and unsharp mask-filtered image stacks. The particles in Figures **3b,c** and **d** were visualized directly in

MATLAB R2014a using the internal surface visualization functions and the VoxelPlotter function from MATLAB File Exchange. All the Avizo and MATLAB visualizations in **Figure 3a,b,c,d** and **f** were acquired from the same aspect to guarantee an appropriate fit between the different image portions. The particle distributions in Figure **3e** were captured from Microsoft Excel 2010, while the box-plots in **Figure 3g** were made using statistical analysis software R 3.1.2. The numbers shown in **Figure 3g** were rounded to at least three significant figures. All the acquired images were appropriately scaled, superimposed and paginated in Inkscape 0.48. The same software was used to redraw the red **Figure 3a** segmentation interfaces thicker in the 2D cross-section images. The interfaces were then superimposed onto the original interface lines acquired from Avizo. In **Figure 3f** the watershed image was hue inverted to give it a distinct orange color.

In **Figure 4a**, ZEN 2011 SP3 (Black Edition) software was used to acquire the CLSM data, which was visualized in Avizo Software 2019.3 using the Ortho Slice module. To label the voxels particles with red and blue colors, the Sieve Analysis module and the Label Analysis module were used to separate the voxel particles into the described volume groups. The Voxelized Rendering module with the Outline enabled was used to present the voxel particles above the grayscale CLSM intensity image inverted with the Invert module. The 2D cross-section image with the segmentation wireframe was visualized using the Segmentation Editor. To capture the **Figure b** and **c** reference image of the five nuclei, Zeiss Axio Vert.A1 light microscope with Zeiss 5x/0,13 inf/0 air objective and Axiocam 105 color camera was used. ZEN 2.3 lite was used to control the camera and capture the image first adjusting the source brightness to enable good exposure, using Auto White Balance and Set Exposure, setting histogram in live view to Min/Max, and the best focus manually achieved was used. The image was saved in 24 bit TIF format for importing into Avizo. The µCT data was presented in similar manner as described above. In all the grayscale images, the lower and upper histogram values were set to present the grayscale ranges with proper contrast without major clipping of the intensity values. The box-plots in **Figure 4d** were made using statistical analysis software R 3.6.2. The numbers shown were rounded to at least three significant figures. All the acquired images were appropriately scaled, superimposed and paginated in Inkscape 0.92. The same software was used to redraw the **Figure 4a,b,** and **c** red segmentation interfaces with thicker lines in the 2D cross-section images. The interfaces were then superimposed onto the original interface lines acquired from Avizo.

Supplementary Note 5: Further discussion about the 3DROQA data processing and quantification results of the µCT-imaged nuclei

Detailed discussion of data processing in the 3DROQA

With the chosen segmentation threshold used to extract the silver signals from the dry-scaffold µCT data (**Supplementary Protocol 3**) in 3DROQA (**Supplementary Protocol 1**), 11,325 objects, referred to as voxel particles, were obtained from the inner FOVs (from two controls and two cytochalasin D-treated samples **Supplementary Table 1**). Similarly, as many as 13,651 voxel particles were obtained from the wet µCT imaging (from three controls and three cytochalasin D-treated samples **Supplementary Table 3**).

To perform statistical analyses on properly processed data, systematic data processing was executed on the obtained particles as follows. To remove small-object noise and to represent the studied shapes by at least a decent number of voxels, dry voxel particles smaller than 15 μm^3 were excluded (**Supplementary Fig. 4**). A 100 μm^3 volume high-pass filter was used for the wet data for the same purpose. The voxel particles were then smoothed out by transforming into polygon particles and later to ellipsoids (**Fig. 3b,c,d** represent various examples found from **Fig. 3e** distribution). In total, 1% of the particles in dry µCT imaging were rejected as hyperboloids (**Supplementary Table 1**), possibly being dividing nuclei or otherwise under-segmented group of nuclei. These hyperboloids were recognized by their negative values returned by the ellipsoid fit function[37]. The volume change in the overall transformation process varied along the relative particle roughness caused by the definite voxel size, for example. This is visible, for example, in the distribution shown in **Figure 3e** as the slightly higher tendency of smaller objects to have small ellipsoid-to-voxels values and the slightly higher tendency of larger objects to have large values, ratios between the volumes of the corresponding ellipsoids and voxel particles. That is why the initial voxel particles were considered to be a more reliable source for the ultimate volume statistics, to better represent the average volume of the measured populations. Data quantization due to the discrete voxels was considered insignificant since it is manifested only at the extreme end of the smallest particles. For example, as many as 110 voxels (in which a single voxel volume is only 0.91%) were used to represent a particle one semi-interquartile range (SIQR) downward from the median volume of the dry control particle population (**Fig. 3e**). Similarly, in the case of wet data, the number of voxels is 68 (in which a single voxel volume is only 1.5%; **Supplementary Fig. 9**). The volume and surface data needed to calculate the sphericity values were acquired from the polygon particles that better represented the morphology of the original nuclei. The objects seemed to be scaled isospatially during the particle transformations (**Fig. 3b,c,d**), which did not affect the sphericity value (proved in **Supplementary Note 6**) or axial ratios calculated from the ellipsoids.

The success of the whole particle transformation process was assessed by the ellipsoid-to-voxels volume ratios that indicate how good a match there is between the initial voxel particle and the corresponding ellipsoid. Example ellipsoids superimposed directly on the voxel particles obtained from the wet µCT imaging are available in **Supplementary Fig. 11**. For most of the particles, the volume ratios were near one per se (**Supplementary Fig. 5** and **12**), and 89% of the dry particles were accepted for the final analysis after the 0.8-1.2 band-pass filtration (**Supplementary Table 1**). Similarly, as many as 92% of the wet particles passed the band-pass filtration (**Supplementary Table 3**). Thus, most of the fits were successful in both dry and

wet cases, which can also be seen from the tight ellipsoid-to-voxels volume ratio distributions around one (**Supplementary Fig. 5** and **12**). No major differences were observed between the data filtrations of the control and cytochalasin D-exposed samples.

Detailed discussion of quantification results of scaffold samples

By comparing the control samples to the cytochalasin D-exposed ones (latter data referred onwards as “experimental”), the cytoskeleton disturbance-related nuclear reduction[38] can be clearly seen from the statistics. In both the dry and wet cases, Wilcoxon-Mann-Whitney shape test p-values indicated statistically very significant differences (p-values $< 2.2 \times 10^{-16}$) and the shape medians became distinctively larger due to the cytoskeleton disturbance (**Fig. 3g** and **Supplementary Fig. 9**). As the large number of analyzed nuclei can produce low p-values without comparably large practical differential significance between the populations, we wanted to further evaluate the distinctiveness between the distributions of the control and experimental values. We considered the populations to be well resolved if the median difference of a considered value was larger than the average SIQR of the two compared groups. In the case of the axis, we considered it convenient to further proportion the median differences to the used voxel dimension. According to these principles, the most prominent shape change was seen in the sphericity values in both dry and wet data. The flatness value manifested the most distinct shape changes from the two axial ratios in both the dry and wet cases. The flatness value was also considered to be the more reliable shape measure from the two axial ratios, since in a few cases the longest axes, used to calculate the elongation values, seemed to overshoot slightly during the ellipsoid fit (an example in **Supplementary Fig. 11e**). In the dry data, all the median shape differences between the control and experimental groups were larger than the corresponding average SIQRs, excluding the elongation value. In the wet data, as expected due to the larger voxel size, the results were not as prominent since the average SIQRs of the shape values covered the differences between the control and experimental medians. According to the absolute shape medians of both the control and the experimental groups, the wet nuclei were rounder in general (**Supplementary Figure 9**). The larger voxels might not represent all the detailed surface irregularities as well, or the wet nuclei might be truly rounder than the dry particles similar to “collapsed sacks” that could express intranuclear precipitate as a rougher surface. However, the lower sampling accuracy of the wet samples should be inherently compensated by the larger nuclei still containing the water.

The other observations, such as the higher signal intensity is seen in the cytochalasin D-treated dry samples, fit to the observed rounding of the experimental nuclei as well (**Fig. 3g**). In addition to the very significant statistical difference (p-value $< 2.2 \times 10^{-16}$), the distinct upper outliers indicate that the cytoskeletal disorganization allowed quite efficient spatial packaging of the antigens in some cases, and perhaps also better labeling access into the nuclei due to the loosened cell structure. This phenomenon was also observable in the wet-imaged samples according to the very low p-value ($< 2.2 \times 10^{-16}$) and the difference of the medians, but otherwise was not as prominent (**Supplementary Fig. 9**). This suggests that the removal of water further aids the packing of all the nuclear matter (the “collapsed sack” comparison discussed previously). The absolute intensity values between the dry and wet data are not directly comparable because the byte scaling for the two reconstruction types was adjusted differently (Methods).

It was expected that the cytoskeleton disturbance-related nuclear deformation would be isovolumetric. For example, we did not consider intracellular reverse osmosis, which is assumed to act more on native nuclei, to be strong enough to see its relief in the experimental samples. This was indicated by the insignificant volume p-values when compared, for example, with the much smaller shape p-values (the difference is at least 14 orders of magnitude large, **Fig. 3g** and **Supplementary Fig. 9**). Furthermore, in the dry samples, the increase was only about 4% from the control median volume, a change over 13 times smaller than the SIQR, and in the wet samples, the change was even less obvious. Still, the volume change between the wet and dry imaging was codirectional, and it is compatible with the protrusion of the shortest axes due to the actin disorganization. The shortest axes are usually the normal ones projection outward from the substrate surface (**Fig. 3a** and **Supplementary Protocol 3**). Although the difference between the small axis medians is only about 77 % from the voxel size of 1.1 µm in the dry imaging, the notable increase of the SIQR extends the distribution to higher values and likely contributes to the distinctively larger flatness values in the experimental group (the SIQRs of the medium and longest axes decreased slightly due to the cytochalasin D exposure). In addition to the very small p-value of the small axial change (p-value $< 2.2 \times 10^{-16}$), these observations indicate slight nuclear swelling in the apical direction due to the disorganization of the cytoskeleton, a similar result to the wet imaging. The growth of the smallest axes compensates the lateral shrinkage visible as clear truncation of the longest axes. The p-value is again extremely small (p-value $< 2.2 \times 10^{-16}$), and the median drop of the longest axes is about two times larger than the voxel size in dry imaging, which we keep practically significant (**Fig. 3g**, a codirectional change with the wet data **Supplementary Fig. 9**). Since the median change of the medium axes is only about 5% from the voxel size in both dry and wet imaging, not to mention the relatively large p-values (the difference is at least 14 orders of magnitude large), the evident changes in the flatness and elongation values likely arise mostly from the changes in the shortest and longest axes, respectively. Thus, by coincidence, the medium axes seemed to function as somewhat static references for assessing the other axes. We did not investigate possible orientation of the lateral axes, typically the medium and longest ones, along the underlying PLA fibers.

The protrusion of the shortest axes due to the actin disorganization was statistically very significant (p-values $< 2.2 \times 10^{-16}$ in both wet and dry data). The shortest axes are usually the ones projection outward from the substrate surface (**Fig. 3a** and **Supplementary Protocol 3**). When inspecting further, the effect did not seem to be as distinct as the changes seen in the shape values, as the small axis median difference was smaller than the average SIQR. These observations indicate statistically significant nuclear swelling in the apical direction due to the disorganization of the cytoskeleton, but the effect is small, especially after taking into account that the difference between the small axis medians is only about 77% from the used voxel size in the dry imaging (0.85 µm increase). The small growth of the smallest axes compensates; however, the lateral shrinkage visible as significant truncation of the longest axes (p-value $< 2.2 \times 10^{-16}$). Considering the practical distinctiveness of the phenomenon, the median difference might be smaller than the average SIQR, but we would like to note that the median drop of the longest axes is about two times larger than the voxel size in dry imaging (2.3 µm decrease), which is considered practically significant (**Fig. 3g**, a codirectional change with the wet data **Supplementary Fig. 9**). Since the median change of the medium axes is only about 5% from the voxel size in both dry and wet imaging, not to mention the relatively large p-values (the difference to the shape p-values is at

least 14 orders of magnitude larger), the evident changes in the flatness and elongation shape values likely arise from the changes in the shortest and longest axes, respectively.

Detailed discussion about quantification of disintegrated dry under-segmented aggregates

It was easy to distinguish and quantify most of the dry µCT-imaged nuclei. However, according to the manual study of the µCT data and optical inspections, erroneous under segmentations of close nuclei in the same voxel entities were known to occur (an example in **Fig. 3f**). These objects were part of the anomalies rejected during the first analytical round (**Fig. 3e** and **Supplementary Figure 5**). We also demonstrated how the distinct nuclear features can still be recognized after using a simple disintegration procedure to break down the dry under-segmented aggregates, including the hyperboloids (**Fig. 3f** and Methods). From the 1,188 initial voxel particles previously rejected as anomalies, 3,218 new particles were extracted for the second round of data filtration (**Supplementary Table 2**). As previously, the new daughter particles were processed through the 15 μm^3 high-pass filter, particle transformations, hyperboloid rejection, and 0.8-1.2 ellipsoid-to-voxels volume ratio filter (**Supplementary Protocol 1**), after which 81% of the new particles were accepted for the reanalysis. Again, the statistically significant bias in all the studied shape properties toward rounder shapes was seen in the cytochalasin D-treated samples (**Supplementary Fig. 8**), an observation that is compatible with the previous results (**Fig. 3g** and **Supplementary Fig. 9**).

With no reason to think otherwise, we assumed that the daughter particles that were disintegrated from the under-segmented aggregates should ideally follow the same statistics as the previous dry data. With this in mind, we further assessed the quality of the new data by comparing the results to the previous observations. Again, in addition to the statistical significance considered in the light of the p-values, to judge the resolution between the control and experimental value distributions, we looked for larger median differences than the corresponding average SIQRs were. This time the condition was only seen in the measurements of the intensity and short axis (**Supplementary Fig. 8**). Thus, the overall differences between the control and experimental groups were not as distinct anymore as they were in the previous dry and wet imaging (**Fig. 3g** and **Supplementary Fig. 9**). The sphericity measurement again manifested the most prominent differences from all the three shape measures, and the flatness measurement was the most sensitive from the two axial ratios. Although the elongation p-value in the disintegration data was again very small according to conventional statistical interpretation (2.14×10^{-7}, **Supplementary Fig. 8**), it was at least nine orders of magnitude larger than previously (**Fig. 3g**). The particle intensities were again higher in the experimental group, indicating the spatial packaging of the nuclear matter and silver precipitates and maybe better labeling access into the disorganized cells.

The reason for the lower overall distinctiveness between the control and experimental value distributions might be in the increased particle roughness caused by the disintegration process. A good example of this is if the rough daughter particles presented in **Figure 3f** are compared to the generally smoother particles acquired directly without the disintegration process (**Figure 3a** and **Supplementary Protocol 3**). Close nuclei likely had overlapping artifacts which falsely enlightened the boundary voxels outside the true nuclei, which is likely the ultimate reason for the

under-segmented aggregates together with the limited resolution of the µCT imaging. The false intermediate voxels, excluding the one voxel wide volume removed during the disintegration process (Methods), became shared between the two daughter particles causing the slight unnatural roughness. This roughness might have also enhanced the previously noted tendency of the longest axes to sometimes overshoot during the ellipsoid fit (example in **Supplementary Fig. 11e**), and therefore contributing to the great deviation in the elongation p-value from the previous dry data. In both the control and experimental groups, the roughness was also manifested in the spreading of the ellipsoid-to-voxels volume ratio distributions (**Supplementary Fig. 7**) as the smooth ellipsoids did not fit so well on to the rougher voxel particles. Other observations distinct to the previous dry data were the notable axial and volume degradations of the daughter particles when. To some extent, the symmetric size reduction in both the control and experimental groups can be explained by the possible size vs. position correlation between recently divided cells. The fresh daughter cells and their nuclei are naturally close to each other, and thus more prone to end up in an under-segmented aggregate, after sharing the common nuclear volume into smaller halves. However, the longest axes of the daughter particles were degraded asymmetrically so much that the median change is now the opposite of what is was; the longest axes are now slightly shorter in the control group. This phenomenon likely had the biggest effect on the deviating elongation p-value when compared to the previous results. Also, the median volumes of the daughter particles dropped to 59% and 87% in control and Cytochalasin D-treated samples, respectively, when compared to the previous dry data. According to these observations, it seems that the more extended and closer reaching control nuclei were more prone to over segmentation. Due to the larger noisy areas the control nuclei cover (**Fig. 3g**), and because the artifacts of neighbors can overlap easier, the control particles are likely more prone to exhibit miss-positioned intensity spikes falsely interpreted as markers during the disintegration procedure. However, the biased tendency to over segment the control particles do not likely cause a false positive interpretation of the results, but it can weaken the sensitivity to detect the overall shape changes in the studied populations as observed. This is because the smaller daughter particles are unlikely more extended than their original particles they were cut from, according to the overall view of the disintegration process (for example, **Fig. 3f**).

Despite the lower data quality, which should be taken into account in future studies, the shape and intensity changes were again reproduced with great statistical significance after the disintegration procedure. The changes in the shape values were in line with the previous dry data, but the volume and axial results should be interpreted with caution. However, we established the disintegration procedure with a rather straightforward and basic approach, and a variety of more sophisticated data processing tools are applicable for further development.

Detailed discussion of quantitative comparison of µCT and CLSM data

When the CLSM and µCT data are compared visually (**Fig. 4a** and **b**,**c**, respectively), the immediate similarities are the nuclear manifestations emerging from the noise and other background anomalies present in both techniques. In both techniques, the obtained voxel particles follow the outlines of their references well in general, despite the 3.3 times larger voxel size in the µCT data. The accepted CLSM voxel particles are smoother and flatter in general, while the µCT voxel particles present noise-induced surface irregularities, and their overall geometries tend to be more round. The strength of the µCT imaging, the capability to capture large FOVs, is apparent in

the 3D perspectives. The µCT landscapes continue well beyond the boundaries of the light-microscope image used to tune the segmentation and reach farther than the CLSM FOV extends. Moreover, the whole imaged volume, with or without liquid immersion, would be reached with the µCT imaging, no matter how optically scattering or absorbing the surrounding media or materials would be, as long as they are not too dense to absorb X-rays efficiently.

If the conventional statistical significance limit of 0.05 and the distributions are considered, the elongation is the least deviating measurement along the whole investigative path (**Fig. 4d**, the p-values obtained from the comparisons to the CLSM data varied between 0.223-0.764). The elongation distributions were somewhat similar to the CLSM data, with or without the higher number of small particles seen in the initial 13 μm^3 high-passed µCT data. Thus, the elongation value could be the least sensitive shape measure, in correlation with the previous observations that the elongation was the least resolved shape measure when the cytoskeletal relaxation in the scaffold samples was investigated (**Fig. 3g, Supplementary Figure 8** and **9**). However, we would like to point out how close the elongation distribution came to the CLSM data after filtering the µCT particles with the longest axis band-pass filter (µCT median only 1.1 % larger; p-value 0.764, **Fig. 4d**). Furthermore, taking into account that the image artifacts should proportionally affect least the largest dimension from which the elongation is calculated and that the cytoskeletal relaxation was captured in the elongation values (**Fig. 3g**, **Supplementary Figure 8** and **9**), we would not like to consider the elongation a useless measurement. Using the **Supplementary Data 2**, we made an additional statistical comparison between the raw µCT data with two types of filtrations: 20.4-28.2 µm longest axis band-pass and 20.4 µm longest axis low-pass, resulting in elongation medians of 0.536 and 0.555, respectively (p-value 0.483). These results indicate a similarity between all the small objects and larger subject nuclei, both sharing about two times longer longest axis than the medium axis, an observation similar to the CLSM elongation measurement (median 0.530). These observations explain the stability of the µCT elongation measurement along the investigative path presented in **Figure 4d**.

The 20.4-28.2 µm longest axis band-passed µCT particles tended to have larger volumes than the CLSM particles (the median volume difference of 9.85 %). Although the p-value of 0.0853 is insignificant in the conventional sense, we expected the µCT particles to be larger due to the coarser voxel size and image artifacts. From the dimensions, the main contributor to the larger observed volume seems to be the shortest axis. After the longest axis band-pass filtration, without or with the NLM image enhancement, the shortest axis deviated from the CLSM data statistically significantly (p-values of 3.13×10^{-5} and 1.82×10^{-5}, respectively). The derivative flatness value also deviated statistically significantly, without or with the NLM image enhancement (p-values of 3.41×10^{-5} and 8.14×10^{-9}, respectively). There might be a couple of reasons for the differences between the shortest axis and the derivative flatness value between the µCT and CLSM data. As seen in the cross-sectional segmentation image of the CLSM data, the *z* axis perpendicular to the imaged substrate surface is not as definitely reproduced as the other axes (**Fig. 4a**). This is the dimension with which the shortest axes of the nuclei similar to lying coins tend to align. This less definite axis could lead to systematic under segmentation in the *z* dimensions as the CLSM fluorescence signals decay into the *z*-blurriness. On the other hand, if the CLSM segmentation in the *z* dimension is considered accurate, the size of the µCT voxel is 16.8 % from the median of the shortest axis. This could result in the conclusion that a single µCT-voxel deviation causes notable bias in the results. However, although the shortest-axis sampling is coarse, it is not useless. The

shortest-axis deviation between the imaging methods is compensated by the CLSM SIQR (1.08 µm) that corresponds to the size of the single µCT voxel (1.1 µm). This means that a single µCT-voxel deviation is about as large as the shortest-axis measurements are spread inherently. It is also notable that the short-axis SIQRs of the longest axis band-passed µCT data are similar to the CLSM data, 1.18 µm and 1.24 µm for the raw and NLM, respectively. This could be considered that the axial error produced in the µCT data is somewhat systematic - a bias caused by the imaging artifacts, error sources which could be compensated using simple correction factors to align the distributions. We recommend considering the possible use of the correction factors on a case-by-case basis, relying on further testing.

We believe a similar superficial inspection in 2D, as demonstrated with reference-based adaptive segmentation principle (**Supplementary Protocol 3**), could be used to obtain adequate settings also for the data filtering in 3D. The properly segmented and filtered µCT and CLSM particles shared similar lateral dimensions, volumes, and part of the shape measures, as shown in **Figure 4d**. This indicates that filtration limits based on the lateral cellular dimensions could be obtained with conventional 2D microscopy, for indirectly filtering data anomalies also in 3D, as the dimensions and other parameters are interconnected through the objects they represent. To compare the characteristics of the µCT and CLSM imaging techniques, we reduced the effect of the biological variability using the tight band-pass filtering based on the lower and upper quartiles of the longest nuclear axis and found correlations with the other measurements as well. However, for purposes other than comparing the characteristics of different imaging techniques, other limits than the tight band-pass based on the lower and upper quartiles of the reference data could be adequate. As an example, if both the reference and the sample do share the same distribution of the subject structures, excluding other particles such as noise, the quartile-based filtration would leave only the 50 % central subject particles in the data. Depending on the purpose and focus of the examination, this might or might not be acceptable. Choosing the proper filtration limits should be considered on a case-by-case basis relying on testing.

Supplementary Note 6: Proof of immunity of sphericity value against varying object size

The *in silico* objects used to represent the µCT-imaged nuclei seemed to be scaled isospatially during the particle transformations (**Fig. 3b,c,d**). The immunity of the sphericity value[39] against isospatial scaling of an object can be easily demonstrated by considering three simple objects. For example, the radius (r) of a perfect sphere is canceled out if the function is reduced, and thus has no effect on the sphericity value:

$$\frac{\pi^{\frac{1}{3}}\,(6\,Volume)^{\frac{2}{3}}}{Area} = \frac{\pi^{\frac{1}{3}}\,\sqrt[3]{6\,Volume}^{\,2}}{Area} = \frac{\pi^{\frac{1}{3}}\,\sqrt[3]{6\,\frac{4}{3}\pi r^3}^{\,2}}{4\pi r^2} = \frac{\pi^{\frac{1}{3}}\,\sqrt[3]{8\pi}^{\,2}\,r^2}{4\pi r^2} = \frac{\pi^{\frac{1}{3}}\,\sqrt[3]{8\pi}^{\,2}}{4\pi} = 1.000$$

The same applies to the edge length (l) of a cube:

$$\frac{\pi^{\frac{1}{3}}\,\sqrt[3]{6\,Volume}^{\,2}}{Area} = \frac{\pi^{\frac{1}{3}}\,\sqrt[3]{6l^3}^{\,2}}{6l^2} = \frac{\pi^{\frac{1}{3}}\,\sqrt[3]{6}^{\,2}\,l^2}{6l^2} = \frac{\pi^{\frac{1}{3}}\,\sqrt[3]{6}^{\,2}}{6} = 0.806$$

The same observation can also be seen with a tetrahedron:

$$\frac{\pi^{\frac{1}{3}}\,\sqrt[3]{6\,Volume}^{\,2}}{Area} = \frac{\pi^{\frac{1}{3}}\,\sqrt[3]{6\,\frac{l^3}{6\sqrt{2}}}^{\,2}}{\sqrt{3}\,l^2} = \frac{\pi^{\frac{1}{3}}\,\frac{l^2}{\sqrt[3]{2}}}{\sqrt{3}\,l^2} = \frac{\pi^{\frac{1}{3}}}{\sqrt{3}\,\sqrt[3]{2}} = 0.671$$

Thus, due to their distinct shapes, all the objects have different sphericity values that describe how efficiently the underlying volume is packed inside the covering surface, no matter what the overall size of the objects is. The least amount of surface area is needed in the case of the perfect sphere, which is why it has a sphericity value of 1.000.

Supplementary Methods

Supplementary Protocol 1: 3DROQA flow chart

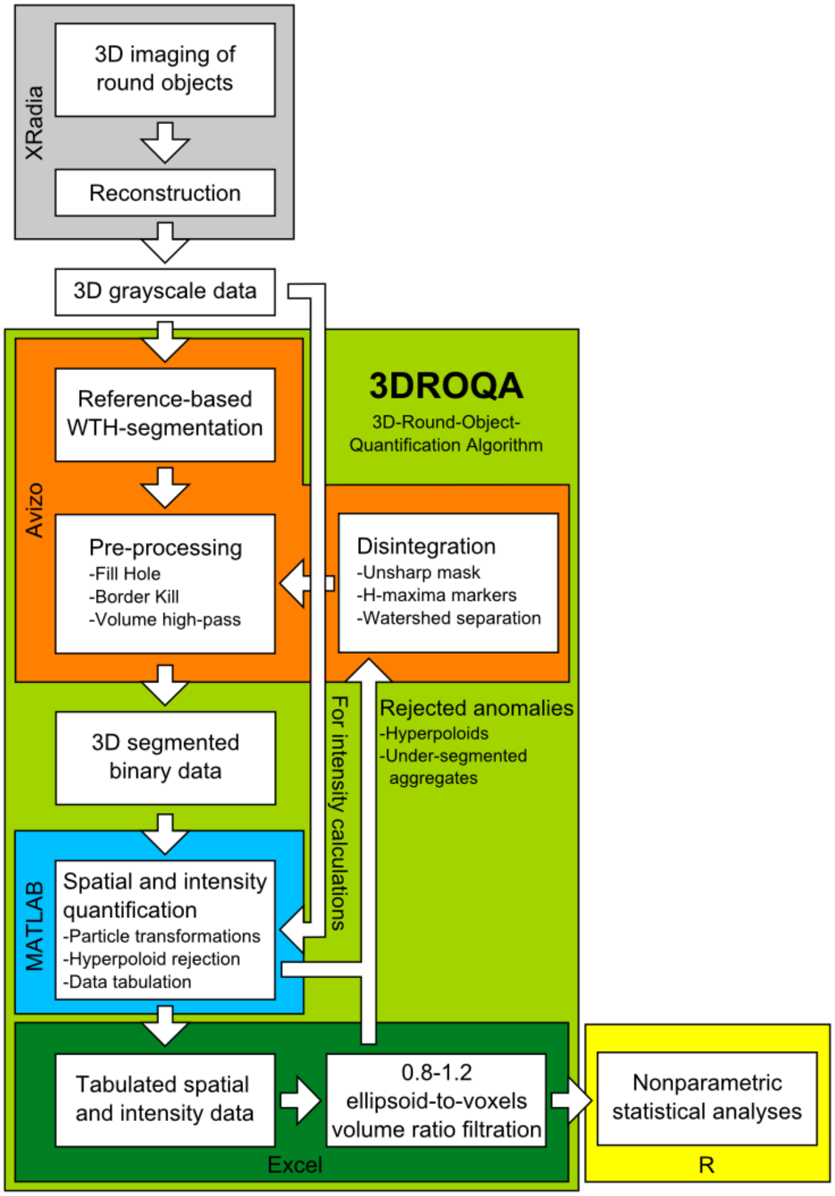

The flow chart above describes the data flow in the 3D-Round-Object-Quantification Algorithm (3DROQA, pale green box). 3DROQA was used to extract and tabulate the spatial and intensity data of the imaged nuclei for the statistical analyses. The other colored boxes show the corresponding software environments in which the different processes were done. The text boxes represent the major steps and the data formats used.

Supplementary Protocol 2: Refractive index adjustment by glycerin titration for optical 3D imaging

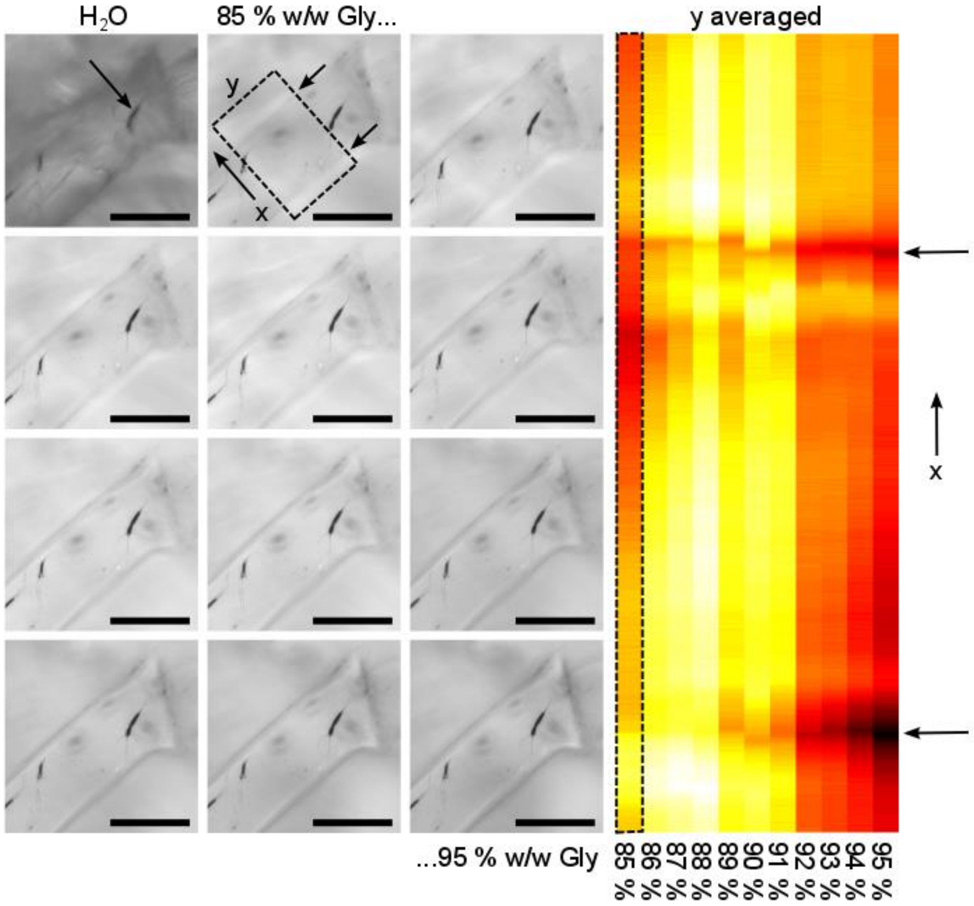

There is a notable refractive index mismatch between water and PLA from about 1.33[(40)] to 1.46[(41)] in the middle of the visible spectrum, respectively. Thus, it is impossible to acquire accurate optical 3D images of the nuclei in water through the PLA fibers (**H_2O**, a focused reference nucleus behind a fiber, indicated by the arrow). Thus, water-diluted glycerin series (**85 – 95 % w/w Gly**) was made to identify the best solution to damp the refraction and clarify the image. The studied series was chosen to include the glycerin concentration that was previously reported in the literature to have a matching refractive index[42] with PLA[41]. The same nucleus, after rinsing and immersing the sample in different glycerin solutions starting from the lowest concentration, was imaged by a light microscope (Evos Fl Auto Cell Imaging, Life Technologies, Thermo Fisher Scientific) with 20x objective. The light intensity was set for each image as high as possible without having any overexposed pixels. From the same location of each image (dashed rectangle), vectors along the direction referred to as x were acquired, and from which all the pixels in the y dimension were averaged to singular values. This intensity data was used to compose a pseudo-color map used to assess the images (right-side panel, a brighter/whiter color refers to higher average intensities). A central group of images emerged in which it was possible to set higher exposure due to the lack of strong focal spots formed by the PLA scaffold. From this group, the central glycerin concertation of **88 % w/w** was considered to be the best option to study the samples by optical means. This

solution allowed the highest and most even amount of light to pass through the scaffold, as the fiber interfaces (indicated by the arrows perpendicular to the x-axis) had the weakest contrast in this image. The steeply changing overall intensities between some of the images (such as between the **91 % w/w** and **92 % w/w**) arose from the discrete light intensity adjustment of the light microscope. Scale bars are 100 µm.

Supplementary Protocol 3: Reference-based adaptive segmentation principle for µCT data and dry reference nuclei

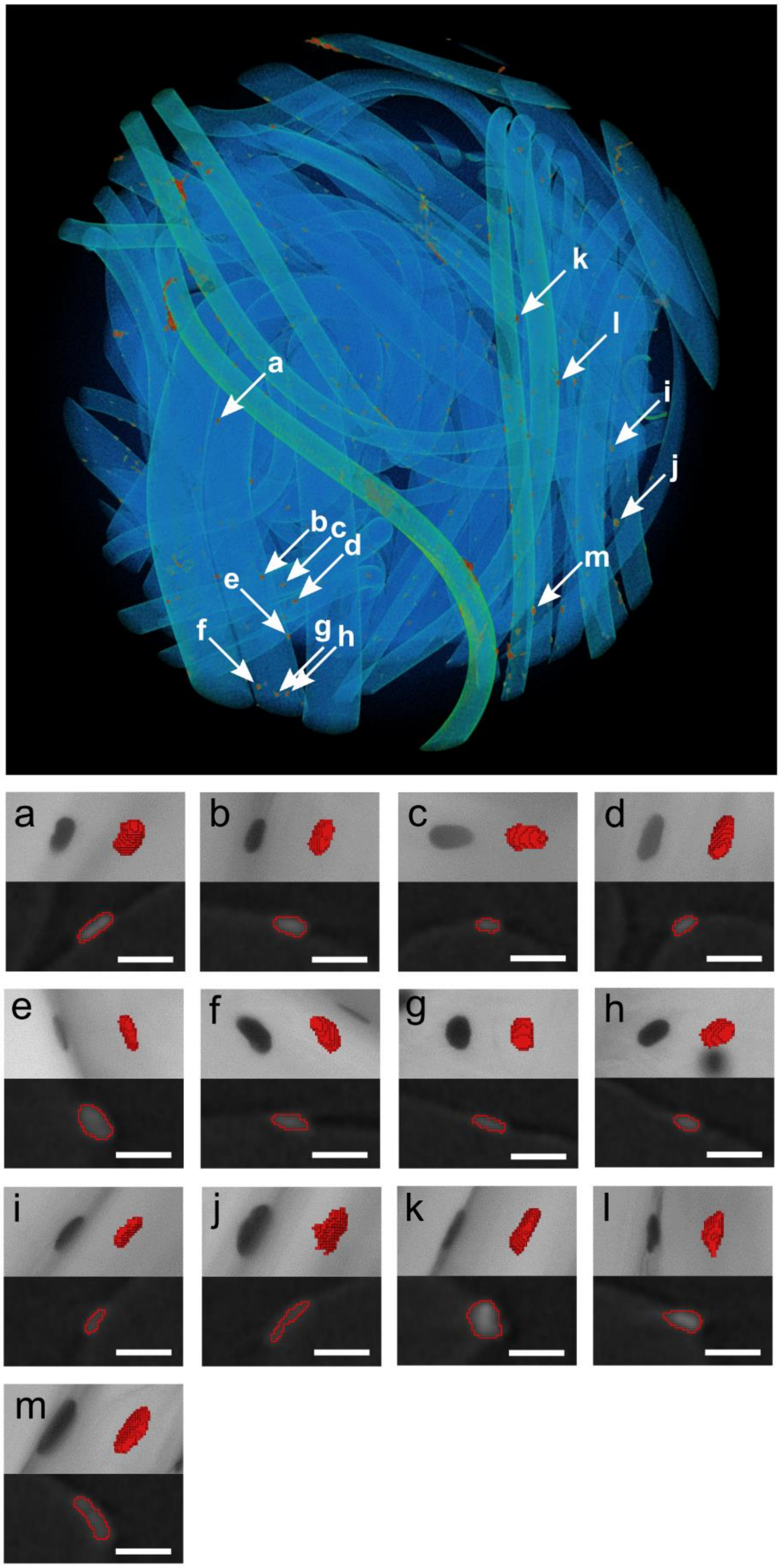

Finding the correct segmentation threshold for the 3DROQA (**Supplementary Protocol 1**) by comparing the optically observed reference nuclei with the µCT data was an important step for quantifying the nuclei (**Fig. 3g**). That is why the access for the 2D optical reference imaging should be enabled, either by imaging the studied cell-cultured application directly or by removing the reference cells prior/after µCT imaging for microscopy. For example, a separate cell-cultured object that fits within the used organ-on-a-chip platform could be used. Objects having at least a similar effective atomic number as the bulk material of the cell-cultured application itself should be used to avoid variations in the overall X-ray attenuation profiles. Once this preliminary adjustment is made, the same threshold value can be used for future samples as long as the experimental parameters do not significantly differ from the initial setup. However, checking the fit of the threshold from time to time is advisable due to the variation related to the longtime aging of X-ray sources and detectors. The demonstrated reference-based adaptive segmentation also helps to exclude a variety of image errors that are weak enough. The image errors might include inappropriate voxels enlightened by possible labeling background, other dense substances, tomographic imperfections such as streaks, ringing, beam hardening, double edges made by sample motion[43], penumbra, partial volume effect[44], and their cooperative actions. Thus, cleaning the µCT data with the reference-based adaptive segmentation can also be useful to visualize the samples more accurately.

The above image represents all the superficial reference nuclei (**a-m**) of a dry control sample used to adjust the segmentation threshold. In the volume-rendered scaffold surface in the uppermost image portion, all the reference nuclei are highlighted with white arrows (more opaque and warmer colors represent higher X-ray attenuation, X-ray pathway aligned along the image plane). Inside the small image boxes below, the upper sections show the voxel entities (red) segmented by the chosen threshold value next to the corresponding nuclei observed with the light microscope (grayscale background images). Arbitrary tomographic cross-sections are shown from the same particles in the lower image sections (grayscale background) on which the segmentation interface at the same depth is superimposed (red frame). The 25 µm scale bars are common to all image portions. The superficial FOV and the **d** nucleus are also visualized in **Supplementary Movie 2**.

By visually judging those nuclei on the PLA fibers not strongly affected by the beam-hardening artifacts (yellowish-green tone around some most superficial, protruding fibers in the volume rendering), the 9/10 voxel particles had a near-perfect fit with their corresponding optical reference nuclei (**a-i**). Only one of these deeper particles had more dissimilar, rougher morphology than its optical reference (**j**), buy it still manifested the same overall dimensions and geometry. The three nuclei on the fibers clearly affected by the beam-hardening artifact also resembled their optical references (**k-m**), but the fit was not as good as most of the nuclei exposed to the more filtered X-rays deeper in the scaffold (**a-i**). Thus, it is important to minimize the spectral variation between the used reference cells and the bulk of the analyzed cells to make the quantification as accurate as possible. For wet samples, this condition is easier to fulfill as the attenuating water itself acts as filtering media and decreases the spectral X-ray variation between the different portions of a sample. However, despite the seen beam-hardening artifacts, after 15 µm^3 high-pass filtering all the 716 found voxel particles, the volume median was found to be 343 µm^3. That is practically the same as was found from the main quantified FOVs (347 µm^3, **Fig. 3g**) positioned deeper into the scaffolds (**Fig. 2**). This demonstrates the applicability of the found threshold, tuned with the superficial µCT imaging, also for the deeper FOVs. Furthermore, if necessary, the weak

beam hardening artifacts could have been easily removed by increasing the X-ray filtration by using an additional filtering material in the X-ray transmission pathway.

It is likely that there was variation in the inspection angles between the light microscopy and μCT reconstruction. However, all the nuclei on the PLA fibers are randomly distributed and they experience the same small angle mismatch. As a result, on average, the size error becomes closer to zero in the observed *xy* dimensions as more nuclei are inspected. Some of the nuclei are tilted so that the seen dimensions become smaller, and for others the dimensions become larger. If enough nuclei are examined, at least a golden mean is enabled where all the voxel particles can be fairly fitted to the microscopy data by using the same threshold. If possible, arranging the reference cells on a flat a surface that is kept perpendicular to the microscope objective, and is visible in the μCT data can be used to minimize the variance between the inspection angles. Including the *z* dimension in the tuning could be helpful, but the fit to optical 3D data also presents difficulties. As demonstrated in **Figure 2a**, the acquired OPT data was badly deteriorated due to the strongly refracting PLA fibers, and the CLSM reconstructions were slightly blurred along the *z* dimension. Therefore, we decided to acquire the reference data in 2D using straightforward and common light microscopy.

Supplementary Protocol 4: Detachment of well-plate-based flat-substrate samples

A metal punch covering the maximal area of the operated well bottoms was chosen to acquire well plate bottom pieces on which the silver-labeled cells were attached (**Fig. 1**). The punch and tweezers were brushed and rinsed with detergent, 70% ethanol, and then with milli-Q-water. The operated well plate was laid onto a soft pile made of cardboard and tissue papers. All wells were covered by a stretched piece of Parafilm to prevent spillage. The target well was exposed with a scalpel, and the punch was laid into the milli-Q-water to touch the bottom. A sharp hit was given with a hammer, and the fractured bottom pieces were quickly collected with the tweezers and placed in fresh milli-Q-water. A laboratory microscope was used to confirm the integrity of the cells before and after the detachment.

Supplementary Protocol 5: Center shift correction principle for µCT reconstructions

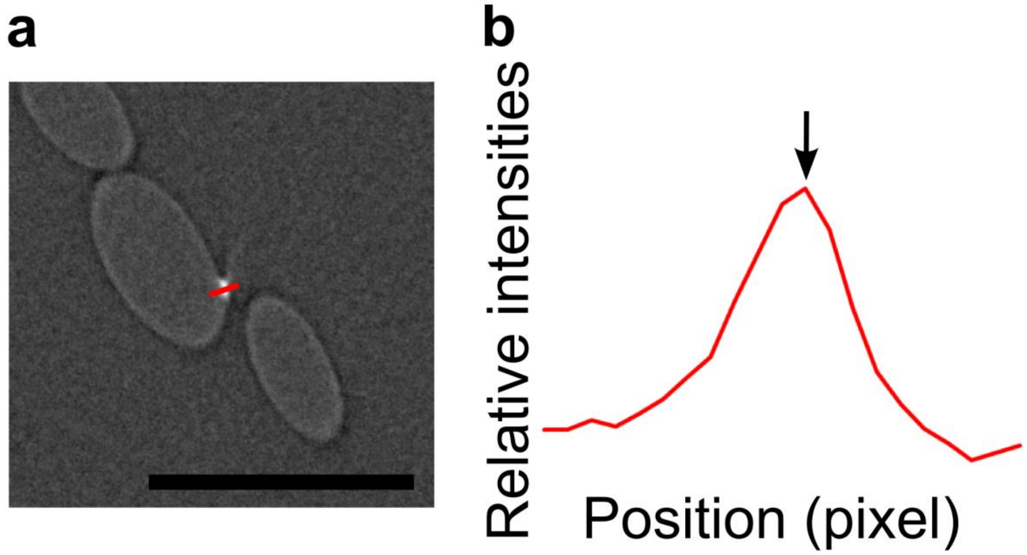

To systematically find the correct center shift correction values for each µCT reconstruction, intensity probe lines (**a**, red line) were drawn over nuclei (bright white feature on a dimmer PLA fiber seen in dry µCT imaging) in preliminary reconstructed cross-sections, an option available in XMReconstructor 8.1. Live intensity plots (**b**) were inspected, and the center shift values between 0.1 intervals were browsed until the highest possible intensity spike (indicated by the arrow) was achieved with the steepest possible edges. The farther the center shift value is from the optimal sharp image, the more blurred the image becomes as the central intensity spike flattens out. Scale bar 200 µm.

Supplementary Figure 1: Indirect antibody-silver labeling principle

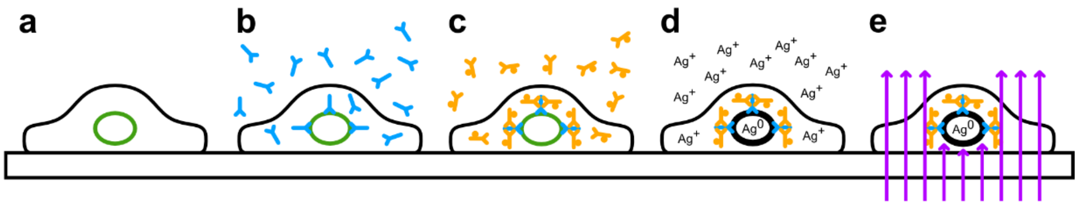

A fixed, permeabilized, and blocked cell ready for the antigen (green) labeling (**a**). Antigen-specific primary antibodies (blue) attach directly to their targets (**b**). Multiple horseradish peroxidase (HRP) functionalized secondary antibodies (orange) attach to singular primary antibodies (**c**). Silver cations (Ag^+) are reduced to metallic silver (Ag^0, thick black line) by the HRP activity (**d**). The labeled cell is subjected to X-rays (purple arrows), which are attenuated by the silver, resulting in a shadow corresponding to the distribution of the antigens (**e**).

Supplementary Figure 2: The entire μCT-imaged flat substrate surfaces of Figure 1

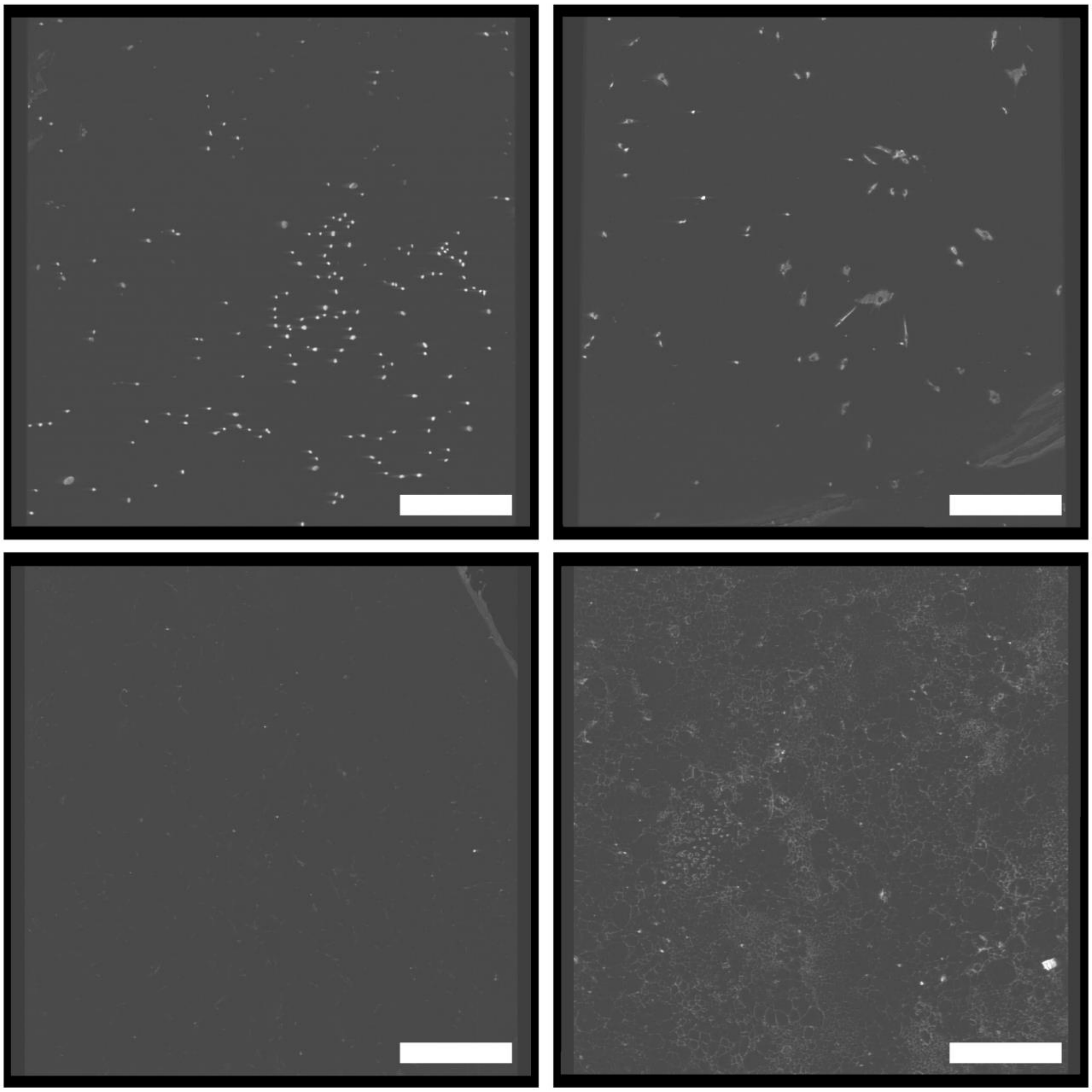

The reconstructions above represent the entire μCT-imaged flat substrate surfaces of lamin (upper left), β-actin (upper right), ATP5α (lower left), and ZO-1 (lower right) cell labeling without histogram adjustments and inversion (close-ups available in **Fig. 1**). The μCT images were formed by showing only the most intensive voxels selected from 100 μm thick stacks perpendicular to the surfaces (maximum intensity projection). The anomalous marks in the substrates are scratches left from the detachment of the well plate bottoms (**Supplementary Protocol 4**). Scale bars are 500 μm.

Supplementary Figure 3: µCT sample-holding syringe and sample stage

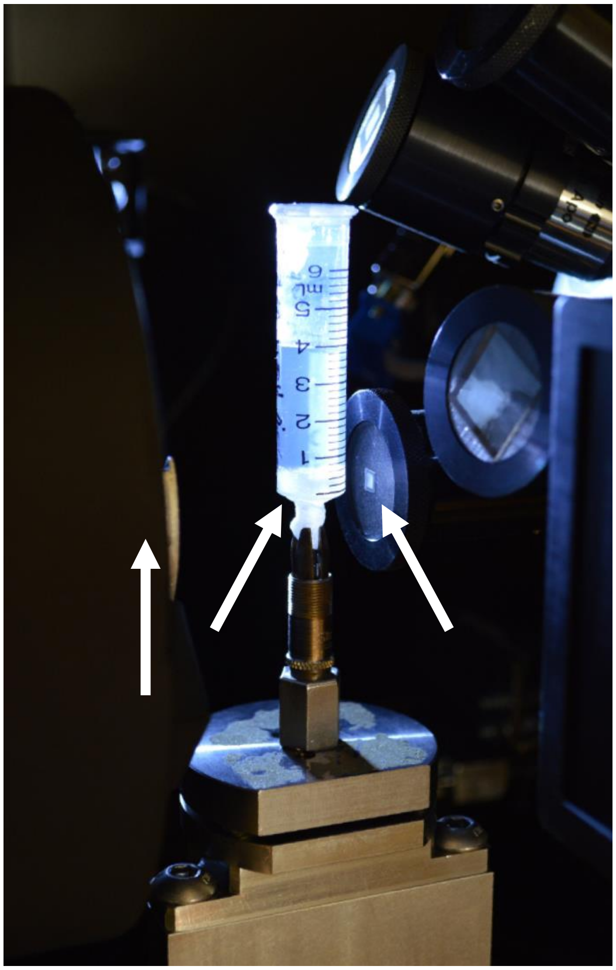

A tissue-engineering scaffold made of PLA immersed in water in the polypropylene syringe (Methods) ready for µCT imaging. The thick coin-shaped sample is resting laterally at the bottom of the syringe (arrow in the middle). The X-ray projections are acquired as the sample is rotated 360 º around the vertical axis. The used X-ray tube source and 10x objective can be seen on the left and the right side of the syringe, respectively (pointed by the corresponding arrows).

Supplementary Figure 4: Whole volume distribution of unfiltered dry µCT-imaged particles

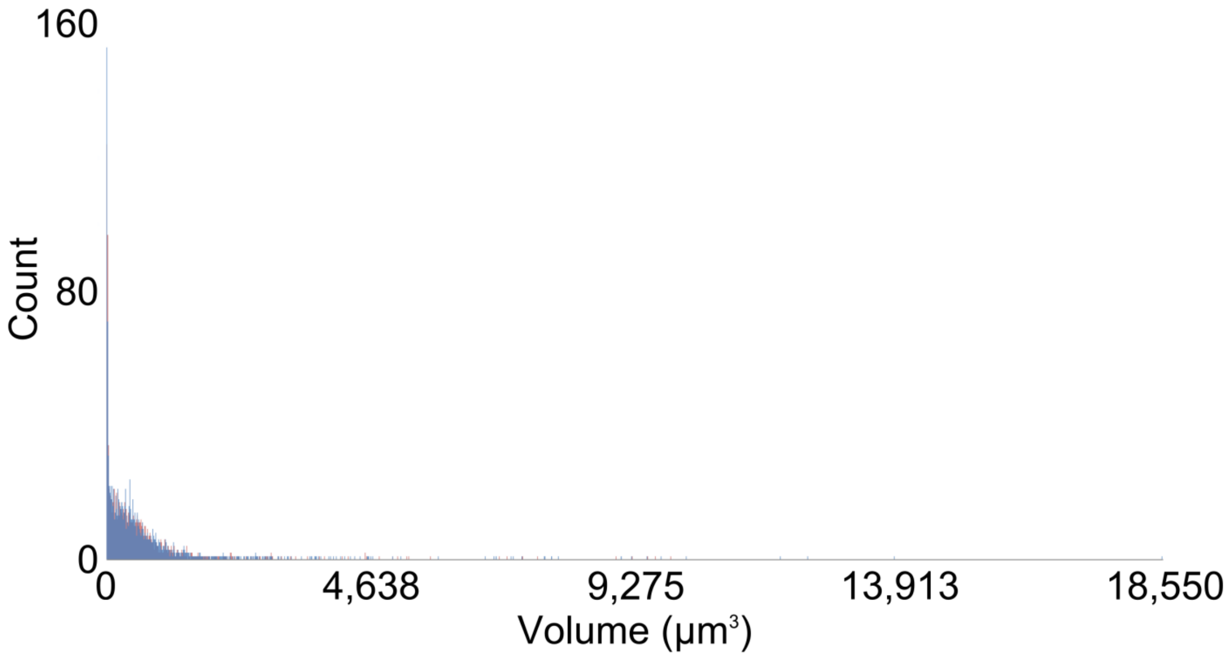

The above histograms show the volume distributions of all the particles found after the white top-hat segmentation in dry µCT-imaged samples. Samples without and with the cytochalasin D treatment are shown in red and blue, respectively. Particles were distributed into one voxel sized bins. Notice the one voxel sized strong noise spike in the data, which was removed along with the other small-object noise using the 15 μm^3 high-pass filtration. The volume distributions of the particles validated for the quantification are shown in **Figure 3g** box-plots.

Supplementary Figure 5: Ellipsoid-to-voxels volume ratio distributions around 1 of dry µCT-imaged particles

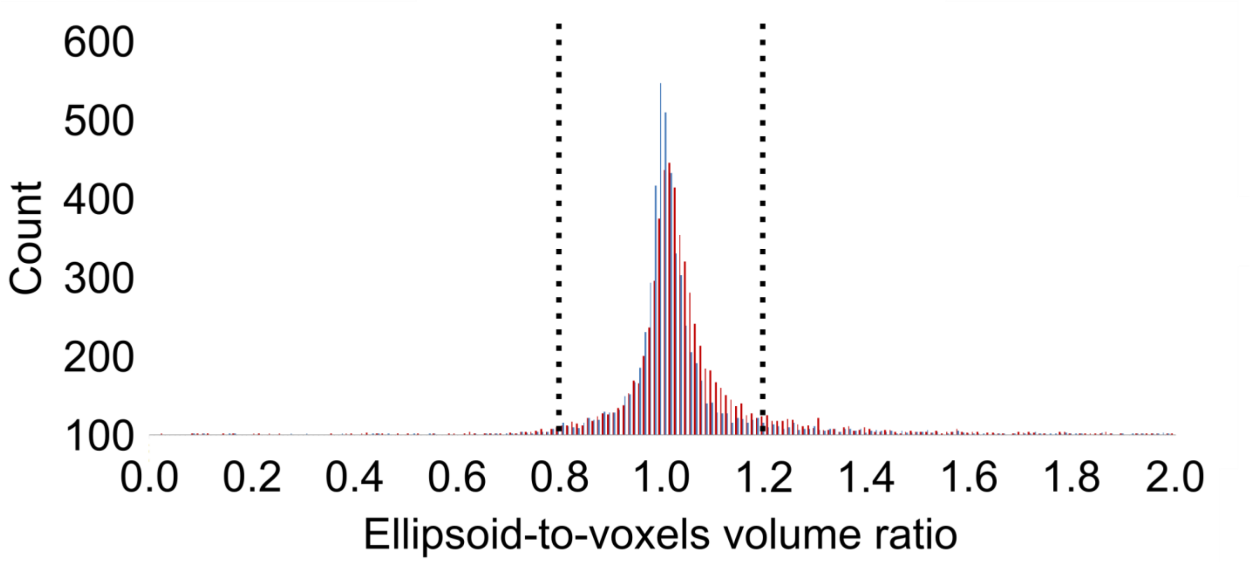

The above histograms show how the ellipsoid-to-voxels volume ratios of the dry µCT-imaged particles were distributed around 1. The particles of the samples without and with the cytochalasin D exposure are represented as red and blue histograms, respectively. Values are divided into 0.01 wide bins, and only the range of 0-2 is shown (see **Fig. 3e** for whole distributions). The used 0.8-1.2 band-pass filter is highlighted by the two vertical dashed lines.

Supplementary Figure 6: Ellipsoid-to-voxels volume ratios vs. volume distributions of nuclei obtained from dry under-segmented aggregates

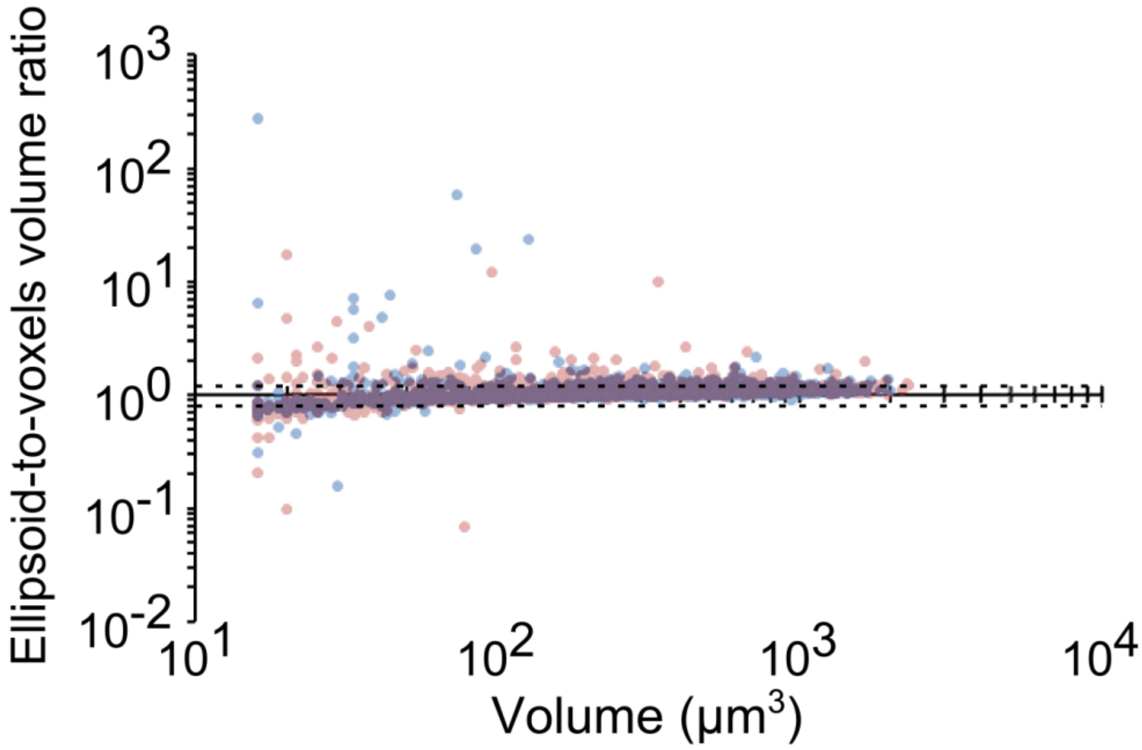

The scatter plot above represents the ellipsoid-to-voxels volume ratio vs. volume distributions of the daughter particles obtained from the dry under-segmented aggregates after 15 μm^3 high-pass filtering. Samples without and with cytochalasin D exposure are represented as red and blue markers, respectively. In total, 83% of the particles were within the range of the volume ratios accepted for the quantification (dashed lines represent the 0.8-1.2 band-pass filter, **Supplementary Table 2**).

Supplementary Figure 7: Ellipsoid-to-voxels volume ratio distributions around 1 of particles extracted from dry under-segmented aggregates

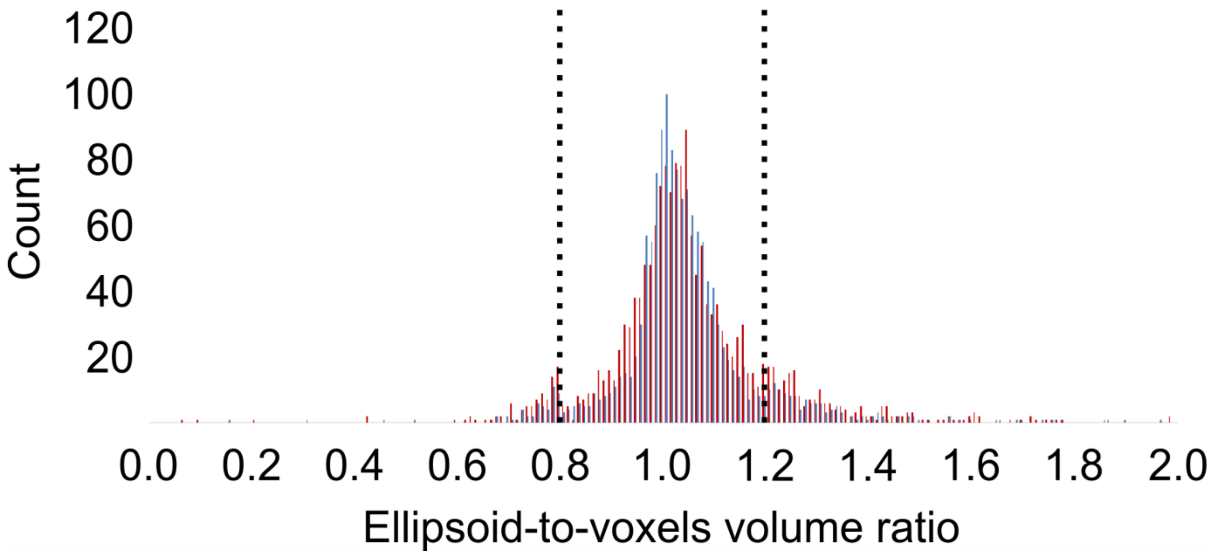

The above histograms show how the ellipsoid-to-voxels volume ratios of the daughter particles obtained from the dry under-segmented aggregates were distributed around 1. The particles without and with the cytochalasin D exposure are represented as red and blue histograms, respectively. Values are divided into 0.01 wide bins, and only the range of 0-2 is shown (see **Supplementary Fig. 6** for whole distributions). The used 0.8-1.2 band-pass filter is highlighted by the two vertical dashed lines.

Supplementary Figure 8: Spatial and intensity quantification of nuclei extracted from dry under-segmented aggregates

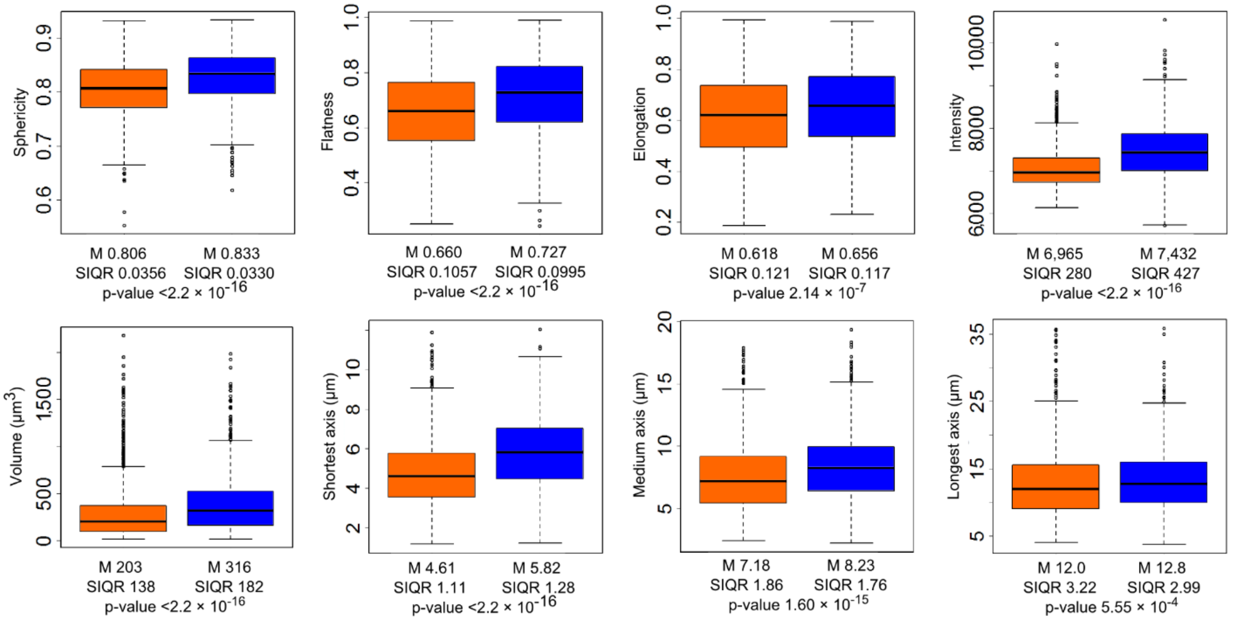

The box-plots above represent the sphericity, flatness, and elongation value, intensity, volume, and axial length distributions of particles extracted from the dry under-segmented aggregates (n = 2 + 2 sample series). The control samples without the cytochalasin D treatment are shown in the boxes on the left (orange) and the exposed ones on the right (blue). The box-plots show the medians (thick center lines), the upper and lower quartiles (horizontal box edges), particles within ±1.5 × interquartile range of the upper and lower quartiles (whiskers), and the rest of the data referred to as outliers (circles). Medians (M) and semi-interquartile ranges (SIQR) are shown for both sample groups below the corresponding distributions together with the p-values of Wilcoxon-Mann-Whitney tests. The sphericity values were calculated from the polygon particles, which consider the found volume of the volume of a perfect sphere, and proportions the corresponding surface area to the surface area of the actual particle (Methods). The flatness value is the ratio between the smallest and medium axes of the fitted ellipsoids, and the elongation proportions the medium axis to the longest one. Thus, all the shape values become one for a perfect sphere. The overall data flow is illustrated in **Supplementary Protocol 1**. The data filtration flow and the number of accepted particles from each sample are described in **Supplementary Table 2**, and the raw data tables are available in **Supplementary Data 2**. Similar results were reproduced three times altogether, more information in **Supplementary Note 5**, **Figure 3,** and **Supplementary Figure 9**. More details about the statistics are given in Methods. Numbers were rounded to at least three significant figures.

Supplementary Figure 9: Spatial and intensity quantification of wet µCT-imaged nuclei

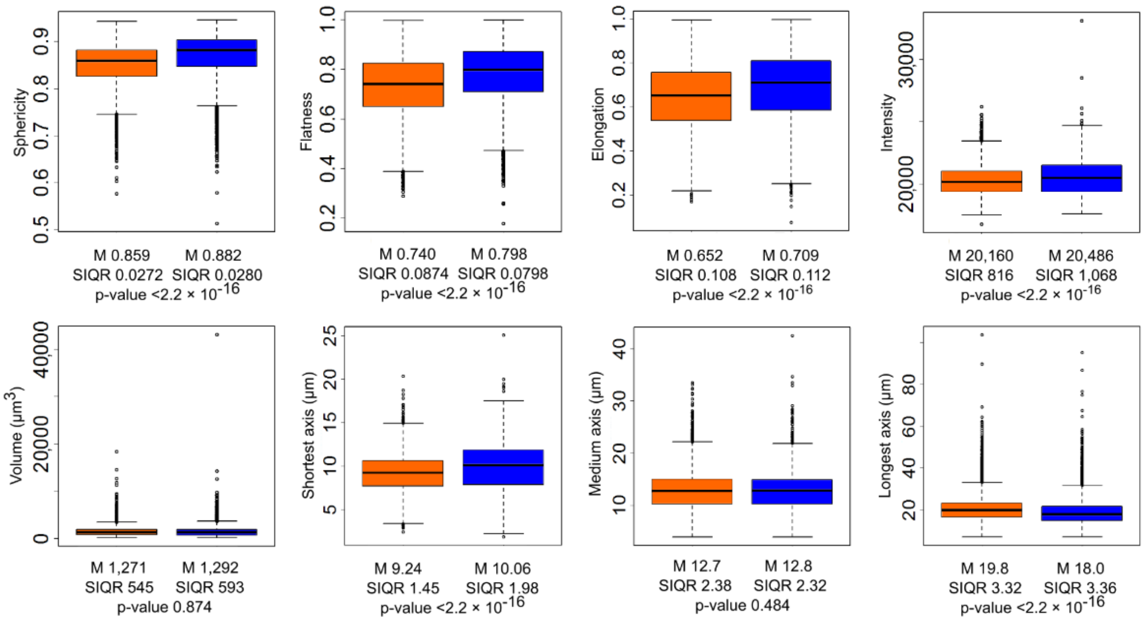

The box-plots above represent the sphericity, flatness, and elongation value, intensity, volume, and axial length distributions of the wet µCT-imaged nuclei (n = 3 + 3 sample series). The control samples without the cytochalasin D treatment are shown in the boxes on the left (orange) and the exposed ones on the right (blue). The box-plots show the medians (thick center lines), the upper and lower quartiles (horizontal box edges), particles within ±1.5 × interquartile range of the upper and lower quartiles (whiskers), and the rest of the data referred to as outliers (circles). Medians (M) and semi-interquartile ranges (SIQR) are shown for both sample groups below the corresponding distributions together with the p-values of Wilcoxon-Mann-Whitney tests. The sphericity values were calculated from the polygon particles, which consider the found volume of the volume of a perfect sphere, and proportions the corresponding surface area to the surface area of the actual particle (Methods). The flatness value is the ratio between the smallest and medium axes of the fitted ellipsoids, and the elongation proportions the medium axis to the longest one. Thus, all the shape values become one for a perfect sphere. The overall data flow is illustrated in **Supplementary Protocol 1**. The data filtration flow and the number of accepted particles from each sample are described in **Supplementary Table 3**, and the raw data tables are available in **Supplementary Data 2**. Similar results were reproduced three times in total, more information in **Supplementary Note 5**, **Figure 3,** and **Supplementary Figure 8**. More details about the statistics are given in Methods. Numbers were rounded to at least three significant digits.

Supplementary Figure 10: White top-hat threshold adjustment for wet µCT imaging

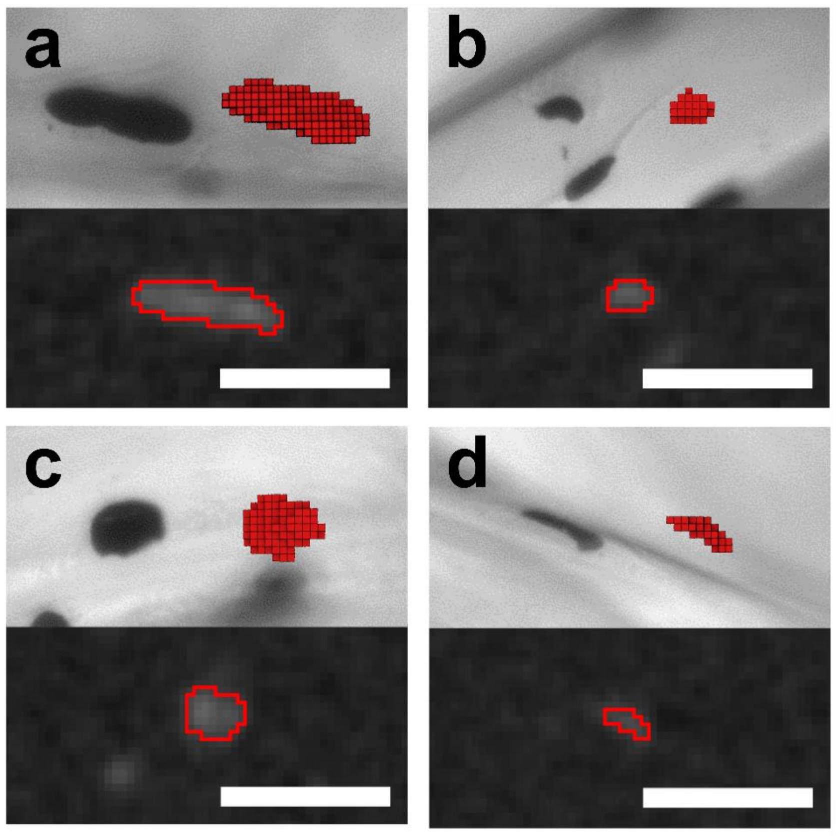

Four examples (**a**, **b**, **c**, and **d**) of the superficial reference nuclei used to adjust the white top-hat segmentation threshold for the two binning wet µCT-imaged samples (more about the tuning method in **Supplementary Protocol 3**). Inside the image boxes, the upper sections show the voxel entities (red) segmented by the chosen threshold value next to the corresponding nuclei observed by a light microscope (grayscale background images). Arbitrary tomographic cross sections are shown from the same particles in the lower sections (grayscale background) on which the segmentation interface at the same depth is superimposed (red frame). Notice that due to the larger voxels and the smaller density difference between the silver and background in the wet imaging, the smallest nuclei were not as well reproduced as in the dry imaging (**Supplementary Protocol 3**). The 50 µm scale bars are common to all image portions.

Supplementary Figure 11: Ellipsoid-to-voxels volume ratios vs. volume distributions with example ellipsoid fits of wet μCT-imaged samples

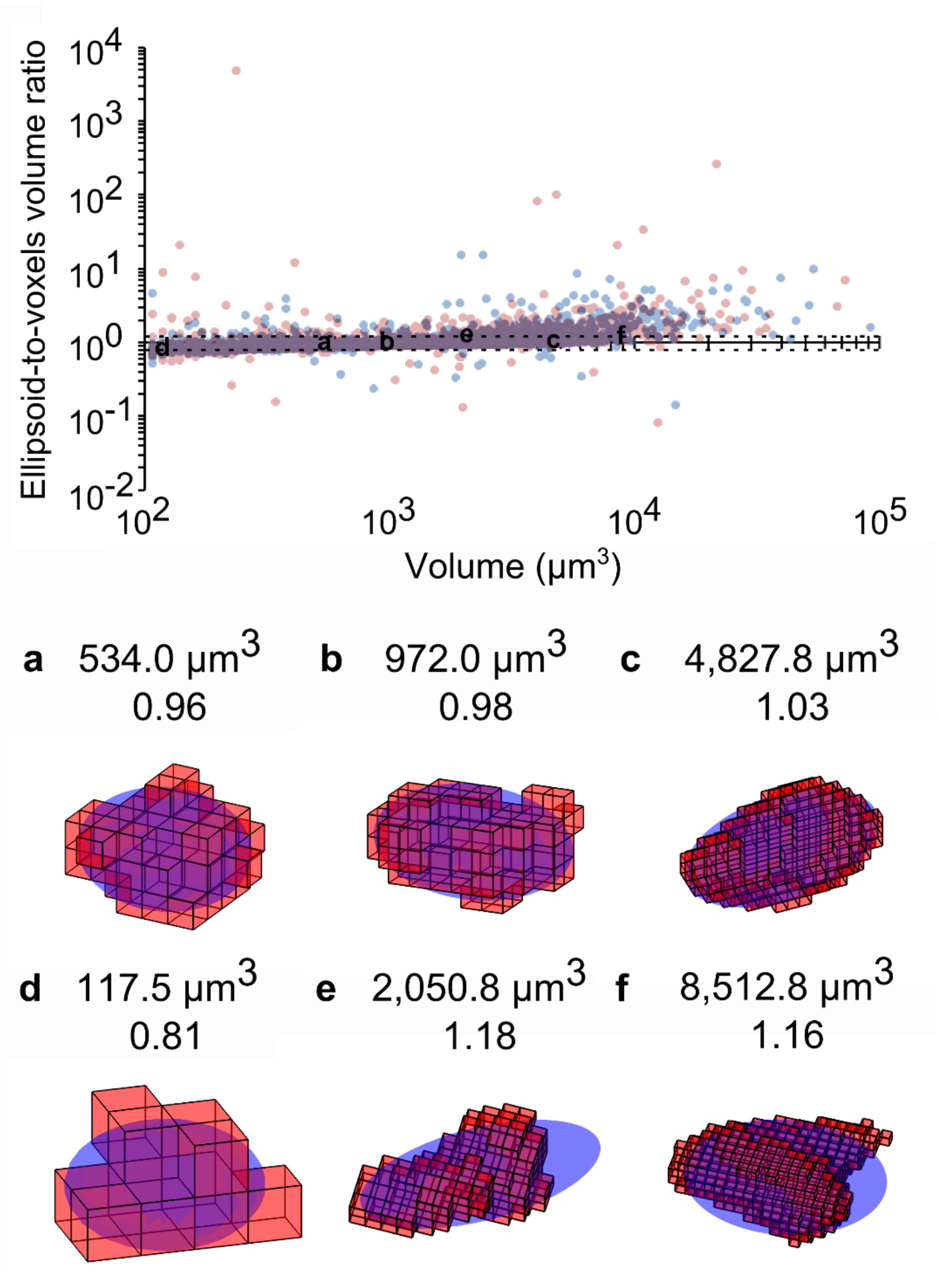

The scatter plot above represents the ellipsoid-to-voxels volume ratio vs. volume distributions of the wet μCT-imaged series after the 100 μm^3 high-pass filtering. Samples without and with cytochalasin D exposure are represented as red and blue markers, respectively. In total, 92% of the particles were within the range of the volume ratios accepted for the quantification (dashed lines represent the 0.8-1.2 band-pass filter, **Supplementary Table 3**). A few accepted example ellipsoid fits are also shown (**a-f**, the voxel particles, and corresponding fitted ellipsoids are shown in red and blue, respectively).

Supplementary Figure 12: Ellipsoid-to-voxels volume ratio distributions around 1 of wet µCT-imaged particles

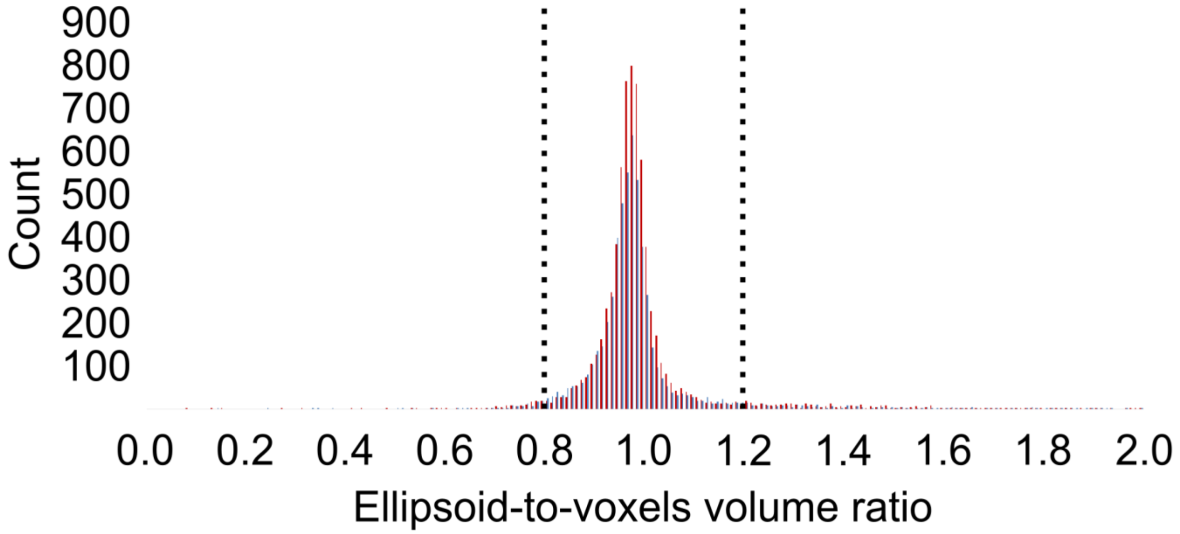

The above histograms show how the ellipsoid-to-voxels volume ratios of the wet µCT-imaged particles were distributed around 1. The particles of the samples without and with the cytochalasin D exposure are represented as red and blue histograms, respectively. Values are divided into 0.01 wide bins, and only the range of 0-2 is shown (see **Supplementary Fig. 11** for whole distributions). The used 0.8-1.2 band-pass filter is highlighted by the two vertical dashed lines.

Supplementary Figure 13: X-ray transmission intensities

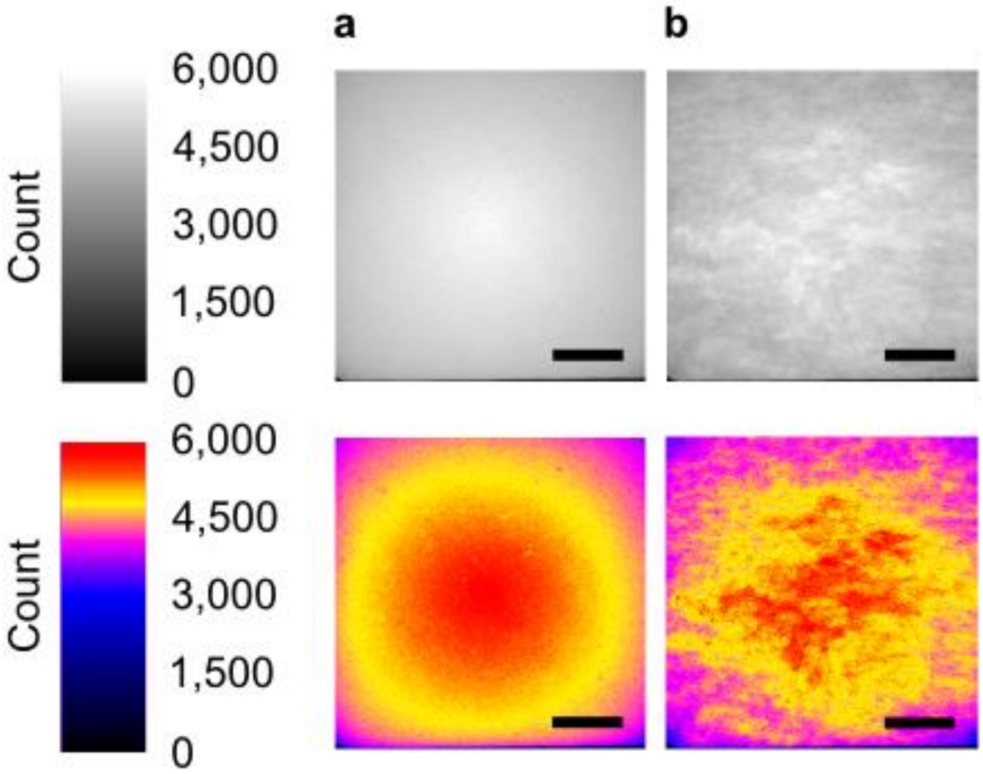

Singular X-ray projection images of wet (**a**) and dry (**b**) PLA scaffolds. Photon count intensities are shown as true-grayscale images (above) and in pseudo-color (below). The 10x objective used had a manufacturing defect seen as the slightly misaligned frame (the dark bottom rim in all the images), which caused a strong, local ringing artifact in the wet reconstructions (**Fig. 2a**). However, it did not affect the other parts of the FOV. This was ensured by the aid of a calibration object and verified by the maintenance experts of the manufacturer. Scale bars are 500 µm.

Supplementary Table 1: Data filtration flow of dry µCT-imaged samples

	Samples without cytochalasin-D treatment			
**Step**	**1**	**2**	**Total**	**% From previous total**
White top-hat segmentation	3,283	2,682	5,965	-
Border kill	3,115	2,510	5,625	94
15 µm^3 high-pass filtration	2,747	2,316	5,063	90
Hyperboloid rejection	2,709	2,289	4,998	99
0.8 < V ellipsoid-to-voxels < 1.2	2,334	2,026	4,360	87
Failed sphericities	*8*	*4*	*12*	*0.3*

	Samples with cytochalasin-D treatment			
**Step**	**1**	**2**	**Total**	**% From previous total**
White top-hat segmentation	2,500	2,860	5,360	-
Border kill	2,424	2,699	5,123	96
15 µm^3 high-pass filtration	2,211	2,407	4,618	90
Hyperboloid rejection	2,180	2,395	4,575	99
0.8 < V ellipsoid-to-voxels < 1.2	1,921	2,212	4,133	90
Failed sphericities	*0*	*4*	*4*	*0.1*

**Step**	**All samples**	**% From previous total**
White top-hat segmentation	11,325	-
Border kill	10,748	95
15 µm^3 high-pass filtration	9,681	90
Hyperpoloid rejection	9,573	99
0.8 < V ellipsoid-to-voxels < 1.2	8,493	89
Failed sphericities	*16*	*0.2*

The above table shows how the initial number of dry µCT-imaged particles were filtered during different data processing steps (Methods) before the final quantification (**Fig. 3g**). The *Failed sphericities* refer to a few cases where the MATLAB code[45] failed to calculate the volume for a polygon particle. Thus, these particles were left without the sphericity value. The dry sample series comprised fewer samples. After the wet µCT imaging, two samples were immersed in glycerin for optical imaging experiments (**Fig. 2** and **Supplementary Protocol 2**).

Supplementary Table 2: Data filtration flow of particles obtained from dry under-segmented aggregates

Step	Samples without cytochalasin-D treatment			
	1	2	Total	% From previous total
Previously rejected particles	413	290	703	-
Disintegration	1,020	704	1,724	245
15 μm^3 high-pass filtration	993	689	1,681	98
Hyperpoloid rejection	979	676	1,655	98
0.8 < V ellipsoid-to-voxels < 1.2	779	550	1,329	80
Failed sphericities	*2*	*1*	*3*	*0.2*

Step	Samples with cytochalasin-D treatment			
	1	2	Total	% From previous total
Previously rejected particles	290	195	485	-
Disintegration	1,043	451	1,494	308
15 μm^3 high-pass filtration	1,033	436	1,469	98
Hyperpoloid rejection	1,027	429	1,456	99
0.8 < V ellipsoid-to-voxels < 1.2	906	347	1,253	86
Failed sphericities	*1*	*1*	*2*	*0.2*

Step	All samples	% From previous total
Previously rejected particles	1,188	-
Disintegration	3,218	271
15 μm^3 high-pass filtration	3,151	98
Hyperboloid rejection	3,111	99
0.8 < V ellipsoid-to-voxels < 1.2	2,582	83
Failed sphericities	*5*	*0.2*

The above table shows how the initial number of the particles obtained from the dry under-segmented aggregates were filtered during different data processing steps (f) before the second round of quantification (**Supplementary Fig. 8**). The *Failed sphericities* refer to a few cases where the MATLAB code[45] failed to calculate the polygon particle volumes. Thus, these particles were left without the sphericity value.

Supplementary Table 3: Data filtration flow of wet µCT-imaged samples

	Samples without cytochalasin-D treatment				
**Step**	**1**	**2**	**3**	**Total**	**% From previous total**
White top-hat segmentation	2,917	2,355	2,226	7,498	-
Border kill	2,889	2,330	2,186	7,405	99
100 µm^3 high-pass filtration	2,723	2,242	2,083	7,048	95
Hyperboloid rejection	2,706	2,230	2,066	7,002	99
0.8 < V ellipsoid-to-voxels < 1.2	2,462	2,071	1,883	6,416	92
Failed sphericities	*3*	*3*	*0*	6	*0.1*

	Samples with cytochalasin-D treatment				
**Step**	**1**	**2**	**3**	**Total**	**% From previous total**
White top-hat segmentation	1,695	2,284	2,174	6,153	-
Border kill	1,672	2,258	2,132	6,062	99
100 µm^3 high-pass filtration	1,583	2,144	1,927	5,654	93
Hyperboloid rejection	1,576	2,131	1,918	5,625	99
0.8 < V ellipsoid-to-voxels < 1.2	1,435	1,953	1,796	5,184	92
Failed sphericities	*1*	*1*	*5*	7	*0.1*

**Step**	**All samples**	**% From previous total**
White top-hat segmentation	13,651	-
Border kill	13,467	99
100 µm^3 high-pass filtration	12,702	94
Hyperboloid rejection	12,627	99
0.8 < V ellipsoid-to-voxels < 1.2	11,600	92
Failed sphericities	*13*	*0.1*

The above table shows how the initial number of the wet µCT-imaged particles were filtered during different data processing steps (Methods) before the final quantification (**Supplementary Fig. 9**). The *Failed sphericities* refer to a few cases where the MATLAB code[45] failed to calculate the polygon particle volumes. Thus, these particles were left without the sphericity value. The wet sample series comprised more samples than the dry series. After the wet µCT imaging, two samples were immersed in glycerin for optical imaging experiments (**Fig. 2** and **Supplementary Protocol 2**).

Supplementary References:

1. Ntziachristos, V. Going deeper than microscopy: The optical imaging frontier in biology. *Nature Methods* **7**, 603–614 (2010).
2. Bouchard, M. B. *et al.* Swept confocally-aligned planar excitation (SCAPE) microscopy for high-speed volumetric imaging of behaving organisms. *Nat. Photonics* **9**, 113–119 (2015).
3. Cella Zanacchi, F. *et al.* Live-cell 3D super-resolution imaging in thick biological samples. *Nat. Methods* **8**, 1047–1050 (2011).
4. Filová, E. *et al.* Analysis and three-dimensional visualization of collagen in artificial scaffolds using nonlinear microscopy techniques. *J. Biomed. Opt.* **15**, 066011-1-066011–7 (2010).
5. Theer, P., Hasan, M. T. & Denk, W. Two-photon imaging to a depth of 1000 µm in living brains by use of a Ti:Al_2O_3 regenerative amplifier. *Opt. Lett.* **28**, 1022–1024 (2003).
6. Horton, N. G. *et al.* In vivo three-photon microscopy of subcortical structures within an intact mouse brain. *Nat. Photonics* **7**, 205–209 (2013).
7. Kobat, D., Horton, N. G. & Xu, C. In vivo two-photon microscopy to 1.6-mm depth in mouse cortex. *J. Biomed. Opt.* **16**, 106014-1-106014–4 (2011).
8. Diao, S. *et al.* Fluorescence imaging in Vivo at wavelengths beyond 1500 nm. *Angew. Chemie - Int. Ed.* **127**, 14971–14975 (2015).
9. Hong, G. *et al.* Through-skull fluorescence imaging of the brain in a new near-infrared window. *Nat. Photonics* **8**, 723–730 (2014).
10. Ding, Z., Ren, H., Zhao, Y., Nelson, J. & Chen, Z. High-resolution optical coherence tomography over a large depth range with an axicon lens. *Opt. Lett.* **27**, 243–245 (2002).
11. Yamanaka, M., Teranishi, T., Kawagoe, H. & Nishizawa, N. Optical coherence microscopy in 1700 nm spectral band for high-resolution label-free deep-tissue imaging. *Sci. Rep.* **6**, 1–8 (2016).
12. Srinivasan, V. J., Radhakrishnan, H., Jiang, J. Y., Barry, S. & Cable, A. E. Optical coherence microscopy for deep tissue imaging of the cerebral cortex with intrinsic contrast. *Opt. Express* **20**, 2220–2239 (2012).
13. Wegel, E. *et al.* Imaging cellular structures in super-resolution with SIM, STED and Localisation Microscopy: A practical comparison. *Sci. Rep.* **6**, 27290 (2016).
14. Winter, P. W. *et al.* Two-photon instant structured illumination microscopy improves the depth penetration of super-resolution imaging in thick scattering samples. *Optica* **1**, 181–191 (2014).
15. Kallai, I. *et al.* Microcomputed tomographyg-based structural analysis of various bone tissue regeneration models. *Nat. Protoc.* **6**, 105–110 (2011).
16. Schneider, G. *et al.* Three-dimensional cellular ultrastructure resolved by X-ray microscopy. *Nat. Methods* **7**, 985–987 (2010).
17. Uchida, M. *et al.* Soft X-ray tomography of phenotypic switching and the cellular response to antifungal peptoids in Candida albicans. *Proc. Natl. Acad. Sci. U. S. A.* **106**, 19375–19380 (2009).
18. Zou, J. *et al.* X-ray microtomographic confirmation of the reliability of CBCT in identifying the scalar location of cochlear implant electrode after round window insertion. *Hear. Res.* **326**, 59–65 (2015).
19. Kerbl, A. *et al.* Micro-CT in cephalopod research: Investigating the internal anatomy of a

sepiolid squid using a non-destructive technique with special focus on the ganglionic system. *J. Exp. Mar. Bio. Ecol.* **447**, 140–148 (2013).
20. Metscher, B. D. MicroCT for developmental biology: A versatile tool for high-contrast 3D imaging at histological resolutions. *Dev. Dyn.* **238**, 632–640 (2009).
21. Metscher, B. D. & Müller, G. B. MicroCT for molecular imaging: Quantitative visualization of complete three-dimensional distributions of gene products in embryonic limbs. *Dev. Dyn.* **240**, 2301–2308 (2011).
22. Thimm, B. W., Hofmann, S., Schneider, P., Carretta, R. & Müller, R. Imaging of cellular spread on a three-dimensional scaffold by means of a novel cell-labeling technique for high-resolution computed tomography. *Tissue Eng. - Part C Methods* **18**, 167–175 (2011).
23. Meyer-Ilse, W. *et al.* High resolution protein localization using soft X-ray microscopy. *J. Microsc.* **201**, 395–403 (2001).
24. Müller, M. *et al.* Nucleus-specific X-ray stain for 3D virtual histology. *Sci. Rep.* **8**, 1–10 (2018).
25. Watling, C. P. *et al.* Novel use of X-ray micro computed tomography to image rat sciatic nerve and integration into scaffold. *J. Neurosci. Methods* **188**, 39–44 (2010).
26. Dorsey, S. M., Lin-Gibson, S. & Simon, C. G. X-ray microcomputed tomography for the measurement of cell adhesion and proliferation in polymer scaffolds. *Biomaterials* **30**, 2967–2974 (2009).
27. Zehbe, R., Goebbels, J., Ibold, Y., Gross, U. & Schubert, H. Three-dimensional visualization of in vitro cultivated chondrocytes inside porous gelatine scaffolds: A tomographic approach. *Acta Biomater.* **6**, 2097–2107 (2010).
28. Mizutani, R., Saiga, R., Takeuchi, A., Uesugi, K. & Suzuki, Y. Three-dimensional network of Drosophila brain hemisphere. *J. Struct. Biol.* **184**, 271–279 (2013).
29. Vågberg, W., Larsson, D. H., Li, M., Arner, A. & Hertz, H. M. X-ray phase-contrast tomography for high-spatial-resolution zebrafish muscle imaging. *Sci. Rep.* **5**, 1–7 (2015).
30. Moosmann, J. *et al.* X-ray phase-contrast in vivo microtomography probes new aspects of Xenopus gastrulation. *Nature* **497**, 374–377 (2013).
31. Sakdinawat, A. & Attwood, D. Nanoscale X-ray imaging. *Nature Photonics* **4**, 840–848 (2010).
32. Larabell, C. A. & Le Gros, M. A. X-ray Tomography Generates 3-D Reconstructions of the Yeast, Saccharomyces cerevisiae, at 60-nm Resolution. *Molecular Biology of the Cell* **15**, 957–962 (2004).
33. Kampschulte, M. *et al.* Nano-Computed Tomography: Technique and Applications. *RöFo Fortschritte auf dem Gebiet der Rontgenstrahlen und der Bildgeb. Verfahren* **188**, 146–154 (2016).
34. Hemberg, O., Otendal, M. & Hertz, H. M. Liquid-metal-jet anode electron-impact x-ray source. *Appl. Phys. Lett.* **83**, 1483–1485 (2003).
35. Kneip, S. *et al.* Bright spatially coherent synchrotron X-rays from a table-top source. *Nat. Phys.* **6**, 980–983 (2010).
36. Berglund, M., Rymell, L., Peuker, M., Wilhein, T. & Hertz, H. M. Compact water-window transmission X-ray microscopy. *J. Microsc.* **197**, 268–273 (2000).
37. Petrov, Y. MathWorks, File Exhance, Ellipsoid Fit. Available at: https://se.mathworks.com/matlabcentral/fileexchange/24693-ellipsoid-fit. (Accessed: 23rd November 2015)
38. Ihalainen, T. *et al.* Differential basal-to-apical accessibility of lamin A/C epitopes in the

nuclear lamina regulated by changes in cytoskeletal tension. *Nat. Mater.* **14**, 1252–1261 (2015).
39. Wadell, H. Volume, Shape, and Roundness of Rock Particles. *The Journal of Geology* **40**, 443–451 (1932).
40. Daimon, M. & Masumura, A. Measurement of the refractive index of distilled water from the near-infrared region to the ultraviolet region. *Appl. Opt.* **46**, 3811–3820 (2007).
41. Hutchinson, M. H., Dorgan, J. R., Knauss, D. M. & Hait, S. B. Optical properties of polylactides. *J. Polym. Environ.* **14**, 119–124 (2006).
42. Hoyt, L. F. New Table of the Refractive Index of Pure Glycerol at 20° C. *Ind. Eng. Chem.* **26**, 329–332 (1934).
43. Davis, G. R. & Elliott, J. C. Artefacts in X-ray microtomography of materials. *Mater. Sci. Technol.* **22**, 1011–1018 (2006).
44. Van Metter, R. L., Beutel, J. & Kundel, H. L. *Handbook of Medical Imaging, Volume 1. Physics and Psychophysics*. *Handbook of Medical Imaging, Volume 1. Physics and Psychophysics* (SPIE Press, 2000). doi:10.1117/3.832716
45. Hannula, M. ThreeDROQA function. (2020). doi:10.5281/zenodo.4008538
