# Supplementary material for: A tube-source X-ray microtomography approach for quantitative 3D microscopy of optically challenging cell-cultured samples
Source: Commun Biol. 2020 Oct 2;3:548. doi: 10.1038/s42003-020-01273-w (PMC7532209; doi:10.1038/s42003-020-01273-w)
Supplement: Supplementary file 3 — Description of additional supplementary items [file 42003_2020_1273_MOESM3_ESM.pdf]

Description of Additional Supplementary Files

File Name: Supplementary Movie 1
Description: Antibody-Silver Generation Observed with Lightmicroscope

File Name: Supplementary Movie 2
Description: Dry Scaffold Surface µCT FOV and Segmentation

File Name: Supplementary Movie 3
Description: Dry Scaffold Center µCT FOV

File Name: Supplementary Movie 4
Description: Dry No-Primary-Control Scaffold Center µCT FOV

File Name: Supplementary Data 1
Description: R Script for Statistical Calculations

File Name: Supplementary Data 2
Description: Tabulated Quantitative Results for µCT and CLSM Data
