# Supplementary material for: A tube-source X-ray microtomography approach for quantitative 3D microscopy of optically challenging cell-cultured samples
Source: Commun Biol. 2020 Oct 2;3:548. doi: 10.1038/s42003-020-01273-w (PMC7532209; doi:10.1038/s42003-020-01273-w)
Supplement: Supplementary file 10 — Reporting summary [file 42003_2020_1273_MOESM10_ESM.pdf]

nature research

Corresponding author(s): Ilmari Tamminen

Last updated by author(s): Sep 1, 2020

Reporting Summary

Nature Research wishes to improve the reproducibility of the work that we publish. This form provides structure for consistency and transparency in reporting. For further information on Nature Research policies, see our Editorial Policies and the Editorial Policy Checklist.

Statistics

For all statistical analyses, confirm that the following items are present in the figure legend, table legend, main text, or Methods section.

n/a	Confirmed	
☐	☒	The exact sample size (n) for each experimental group/condition, given as a discrete number and unit of measurement
☐	☒	A statement on whether measurements were taken from distinct samples or whether the same sample was measured repeatedly
☐	☒	The statistical test(s) used AND whether they are one- or two-sided<br>*Only common tests should be described solely by name; describe more complex techniques in the Methods section.*
☒	☐	A description of all covariates tested
☐	☒	A description of any assumptions or corrections, such as tests of normality and adjustment for multiple comparisons
☐	☒	A full description of the statistical parameters including central tendency (e.g. means) or other basic estimates (e.g. regression coefficient) AND variation (e.g. standard deviation) or associated estimates of uncertainty (e.g. confidence intervals)
☒	☐	For null hypothesis testing, the test statistic (e.g. F, t, r) with confidence intervals, effect sizes, degrees of freedom and P value noted<br>*Give P values as exact values whenever suitable.*
☒	☐	For Bayesian analysis, information on the choice of priors and Markov chain Monte Carlo settings
☒	☐	For hierarchical and complex designs, identification of the appropriate level for tests and full reporting of outcomes
☒	☐	Estimates of effect sizes (e.g. Cohen's d, Pearson's r), indicating how they were calculated

Our web collection on statistics for biologists contains articles on many of the points above.

Software and code

Policy information about availability of computer code

Data collection	Multiple software were used to execute various parts of the research. Like many other details requested in this form, accurate software information is available in the manuscript and supplementary material. To have good overall view on the software used for the 3D quantification, please see the Supplementary Protocol 1. The exact software versions are available in the Methods section also for the different imaging techniques. Details about the image composition in the main article figures including the used graphical software are reported in the Supplementary Note 4.
Data analysis	See above.

For manuscripts utilizing custom algorithms or software that are central to the research but not yet described in published literature, software must be made available to editors and reviewers. We strongly encourage code deposition in a community repository (e.g. GitHub). See the Nature Research guidelines for submitting code & software for further information.

Data

Policy information about availability of data

All manuscripts must include a data availability statement. This statement should provide the following information, where applicable:

- Accession codes, unique identifiers, or web links for publicly available datasets
- A list of figures that have associated raw data
- A description of any restrictions on data availability

Like stated in the manuscript, the MATLAB code that was used to run the automatic operations of the 3DROQA (Supplementary Protocol 1) is provided in: Hannula, M. ThreeDROQA function. (2020). doi:10.5281/zenodo.4008538. The data ready for the statistical analysis, exported and tabulated by the MATLAB, is provided in the Supplementary Data 2. The used R script file, used to execute the statistical analysis, is provided in the Supplementary Data 1. If requested, we are happy to provide also the original raw 3D uCT data files for the reviewers and readers. The Figure 1 uCT images are also available without histogram adjustment and grayscale

inversion in full scale in the Supplementary Figure 2. More details about the main-article-image compositions are given in the Supplementary Note 4.

Field-specific reporting

Please select the one below that is the best fit for your research. If you are not sure, read the appropriate sections before making your selection.

☒ Life sciences ☐ Behavioural & social sciences ☐ Ecological, evolutionary & environmental sciences

For a reference copy of the document with all sections, see nature.com/documents/nr-reporting-summary-flat.pdf

Life sciences study design

All studies must disclose on these points even when the disclosure is negative.

Sample size

No preliminar sample-size calculations were made. The exact number of samples, FOVs, and acquired particles in silico are described in the paper.

Data exclusions

It was assumed that the raw 3D data will include imperfections with various origins such as imaging noise. Systematic data filtration, explained in detail in the manuscript and supplementary data, was used to remove one-to-a-few-voxel objects (much smaller than the observed median nuclear sizes were), and incomplete objects cut by the edge of the FOV. According to the reported data-quality indicators, other data anomalies were excluded from the analysis as well. Part of these anomalies were under-segmented close nuclei, which were later reprocessed and disintegrated in silico for the second round of analysis. After the disintegration, the statistical analysis applied on the new separate nuclei reproduced similar results in line with the original data. Data-filtration flow and the number of objects rejected at various steps are tabulated in detail in Supplementary Table 1, 2 and 3. Special attention was paid for cleaning the Figure 4 CLSM data to ensure that it represented only true nuclei well-resolved used as reference, the data exclusions described in detail in methods.

Replication

Not only the examined sub-cellular phenomenon was captured with the used uCT analysis more efficiently than we expected, but the same results were succesfully reproduced with rather different analytical setups. The same clear nuclear rounding, induced by the cytoskeleton-disturbing agent, was observed in both wet and dry imaged samples, and in the in silico disintegrated under-segmented nuclei. All the observations within and across the experimental series were coherent and expected. Furthermore, in Figure 4 the visual and quantitative similarities and dissimilarities of the µCT imaged nuclei with CLSM imaged nuclei in 3D were assessed in detail. These observations help to define the accuracy and reproducibility of the demonstrated uCT imaging and analysis method.

Randomization

All the samples were prepared and handled in parallel with great control on reproducibility. Half of the scaffold samples were randomly chosen for the cytochalasin-D exposure. After the wet imaging, two randomly chosen samples were needed for optical imaging: one for optimizing the refractive-index matching water-glycerin solution, and one that was directly put into the optimized solution for the actual 3D optical imaging. Thus, the first wet imaging experiment included 3 + 3 samples; and the following dry experiment included 2 + 2 samples. Similarly, for the Figure 4 experiment the flat-substrate samples were randomly chosen for the µCT and CLSM labeling and imaging. The acquired FOVs were randomly positioned on cellular parts of the samples avoiding the overlapping of the FOVs and counting of the same cells multiple times.

Blinding

To avoid additional layer of complexity and possibility for human error, we did not see the use of blinding to be necessary for our experiments. Many of the data processing steps were automatized with computer algorithms.

Reporting for specific materials, systems and methods

We require information from authors about some types of materials, experimental systems and methods used in many studies. Here, indicate whether each material, system or method listed is relevant to your study. If you are not sure if a list item applies to your research, read the appropriate section before selecting a response.

Materials & experimental systems

n/a	Involved in the study	
☐	☒	Antibodies
☐	☒	Eukaryotic cell lines
☒	☐	Palaeontology and archaeology
☒	☐	Animals and other organisms
☒	☐	Human research participants
☒	☐	Clinical data
☒	☐	Dual use research of concern

Methods

n/a	Involved in the study	
☒	☐	ChIP-seq
☒	☐	Flow cytometry
☒	☐	MRI-based neuroimaging

Antibodies

Antibodies used

The following dilutions, as recommended by the manufacturers for conventional antibody labeling, were used for primary antibodies: 1:100 anti-β-actin (C4 sc-47778, Santa Cruz Biotechnology); 1:200 anti-lamin (A + C [131C3] ab8984, Abcam); 1:1000 anti-ATP5α (ab14748, Abcam); 1:100 anti-ZO-1-1A12 (33-9100, Invitrogen). The used secondary antibody for silver labeling was provided within a ready-to-use kit (HRP functionalized secondary antibody labeling kit, MP-7402, Vector Laboratories). The secondary antibody used for

	the fluorescent CLSM labeling was 1:200 diluted A488 goat anti-mouse (IgG (H+L) highly cross-adsorbed, Invitrogen).
Validation	All the used antibodies were applicable on human cells as stated by the manufacturers.

Eukaryotic cell lines

Policy information about cell lines

Cell line source(s)	Human adipose stem cells (hASCs) were isolated from an adipose tissue sample obtained surgically from a 50-year old female donor. Caco-2 cells were acquired from American Type Culture Collection, ATCC (#HTB-37).
Authentication	Cell lines used were not authenticated.
Mycoplasma contamination	The cell lines were routinely tested for mycoplasma with negative results.
Commonly misidentified lines (See ICLAC register)	Using the search terms "ASC", "adipose", "CaCo", or "colorectal adenocarcinoma" did not produce any relevant results from the Cross-Contaminations v10 list obtained from the website of The Register of Misidentified Cell Lines - ICLAC.
